# Chromosome‐based survey sequencing reveals the genome organization of wild wheat progenitor *Triticum dicoccoides*

**DOI:** 10.1111/pbi.12940

**Published:** 2018-06-13

**Authors:** Bala Ani Akpinar, Sezgi Biyiklioglu, Burcu Alptekin, Miroslava Havránková, Jan Vrána, Jaroslav Doležel, Assaf Distelfeld, Pilar Hernandez, The IWGSC, Hikmet Budak

**Affiliations:** ^1^ Department of Plant Sciences and Plant Pathology Cereal Genomics Lab Montana State University Bozeman MT USA; ^2^ Centre of the Region Haná for Biotechnological and Agricultural Research Institute of Experimental Botany Olomouc Czech Republic; ^3^ Department of Molecular Biology and Ecology of Plants Faculty of Life Sciences Tel Aviv University Tel Aviv Israel; ^4^ Instituto de Agricultura Sostenible (IAS) Consejo Superior de Investigaciones Científicas (CSIC) Cordoba Spain; ^5^ International Wheat Genome Sequencing Consortium Bethesda MD USA

**Keywords:** wild emmer wheat, chromosome sorting, hexaploid wheat, next‐generation sequencing, comparative genomics

## Abstract

Wild emmer wheat (*Triticum turgidum* ssp. *dicoccoides*) is the progenitor of wheat. We performed chromosome‐based survey sequencing of the 14 chromosomes, examining repetitive sequences, protein‐coding genes, miRNA/target pairs and tRNA genes, as well as syntenic relationships with related grasses. We found considerable differences in the content and distribution of repetitive sequences between the A and B subgenomes. The gene contents of individual chromosomes varied widely, not necessarily correlating with chromosome size. We catalogued candidate agronomically important loci, along with new alleles and flanking sequences that can be used to design exome sequencing. Syntenic relationships and virtual gene orders revealed several small‐scale evolutionary rearrangements, in addition to providing evidence for the 4AL‐5AL‐7BS translocation in wild emmer wheat. Chromosome‐based sequence assemblies contained five novel miRNA families, among 59 families putatively encoded in the entire genome which provide insight into the domestication of wheat and an overview of the genome content and organization.

## Introduction

Wheat, a major cereal food crop, is rich in carbohydrates, proteins and minerals and is grown on over 220 million hectares of land worldwide (Henry *et al*., 2016; Mayer *et al*., 2014). However, yield trends show increasing instability worldwide, particularly across developing regions, where wheat represents a staple food source (Iizumi *et al*., 2014). The formation of *T. aestivum* occurred through at least two spontaneous hybridizations accompanied by whole‐genome duplications. Several hundred thousand years ago, a spontaneous hybridization between the diploid A‐genome progenitor, *Triticum urartu* (2*n* = 2*x* = 14, AA), and the unknown B‐genome progenitor, a close relative of extant *Aegilops speltoides* (BB), led to the formation of allotetraploid *Triticum turgidum* (2*n* = 4*x* = 28, AABB), which eventually gave rise to bread wheat after a subsequent hybridization with the diploid D‐genome progenitor *Aegilops tauschii* (2*n* = 2*x* = 14, DD) (Marcussen *et al*., 2014). While the allotetraploid wheat was also domesticated and is being cultivated as durum wheat, wild populations that diverged into subspecies continue to exist. Among these wild populations is wild emmer wheat, *Triticum turgidum* ssp. *dicoccoides* (2*n* = 4*x* = 28, AABB), the wild relative of durum wheat. *T. dicoccoides* populations show remarkable genetic diversity for traits such as grain micronutrient content, abiotic stress tolerance and biotic stress resistance (Budak *et al*., 2013b; Ergen and Budak, 2009; Ergen *et al*., 2009). For instance, *T. dicoccoides* genotype TR39477 exhibits outstanding tolerance to drought, whereas another genotype, TTD‐22, is highly susceptible to this stress (Ergen and Budak, 2009; Ergen *et al*., 2009). Such genetic diversity should allow researchers to explore the molecular basis of these traits and discover favourable alleles for breeding. In addition, the direct ancestry of *T. dicoccoides* to bread wheat facilitates gene transfer through viable crosses, making it a promising resource for wheat improvement. Despite its great potential, this resource remains largely untapped (Akpinar *et al*., 2015a; Budak *et al*., 2013a; Ergen and Budak, 2009; Nevo and Chen, 2010).

The draft whole‐genome sequence of bread wheat was published a couple of years ago (Mayer *et al*., 2014), following publication of the genome sequences of the A‐ and D‐genome progenitors (Jia *et al*., 2013; Ling *et al*., 2013). While a much improved assembly was recently released for bread wheat (Zimin *et al*., 2017), to fully exploit these genomic resources for wheat improvement, it will be crucial to unlock the contents of the rich gene pools of its wild relatives. To this end, next‐generation sequencing of the 5B chromosome of *T. dicoccoides* was recently performed, providing the first in‐depth insights into its genome (Akpinar *et al*., 2015b). Finally, a whole‐genome assembly was published for *T. dicoccoides* genotype Zavitan (Avni *et al*., 2017), which provided valuable insight into the wild tetraploid wheat genome. These genomic resources will become much more useful as they accumulate including additional genotypes and relatives.

Here, we report next‐generation sequencing of all 14 *T. dicoccoides* chromosomes isolated by flow cytometry, which enabled us to explore the repetitive sequence landscape, protein‐coding genes, putative miRNA/target pairs and tRNA genes in this species and its syntenic relationships with related grasses. Analysis of this large‐scale genome data based on individual chromosomes allowed us to compare the subgenomes of *T. dicoccoides*. This study expands our knowledge of the wild emmer genome at the nucleotide level, paving the way for comparative analyses and molecular marker discovery that should facilitate the identification, cloning and transfer of favourable alleles for wheat improvement.

## Results

### Bi‐parametric flow cytometry provides access to individual *T. dicoccoides* chromosomes

To circumvent genome complexity of tetraploid *T. dicoccoides*, all 14 chromosomes were individually isolated by flow cytometry from two genotypes, based on bi‐parametric analysis of GAA microsatellite content and DAPI fluorescence intensity, as described earlier (Akpinar *et al*., 2015b). Chromosomal DNA amplified from flow‐sorted chromosomes was sequenced to a depth of 39–58 × , assuming that chromosome sizes are similar to those estimated for *Triticum durum* cv. Timilia (Venora *et al*., 2002). After exclusion of ultra‐short contigs of <200 bases that likely represent collapsed repeats (Mayer *et al*., 2014; Simpson *et al*., 2009), the *de novo* assembly built from >480 Gb sequence data was mapped to the recently published genome of *T. dicoccoides* genotype Zavitan (Avni *et al*., 2017) to eliminate any impurities and contamination from organellar genomes and/or rDNA sequences. Neighbouring scaffolds of the *de novo* assembly mapping to the appropriate Zavitan chromosomes closer than a distance of 50 nucleotides were merged with ‘N's to build chromosomal super‐scaffolds. On average, chromosomal super‐scaffolds represented ~32%–78% of their respective Zavitan chromosomes. These reference‐guided final super‐scaffolds, referred as ‘chromosome assemblies’ hereafter, ranged from 288.3 to 700.4 Mb in total length, with N50 values of 1092–4938 bp. (Table 1).

**Table 1:** Summary of sequencing and assembly statistics for *T. dicoccoides* chromosomes

	Raw data				Assembly					Repetitive sequences (%)
	Estimated size (Mbp)^a^	Purity in flow‐sorted fraction (%)	Sequences (Gbp)	Coverage (x‐fold)	k‐mer for assembly	Total no. of final contigs and clusters (>200 bp)	Assembled sequences (Mbp)	N50 (bp)	% representation of respective Zavitan chromosomes	
Tdic1A	657.6	92	38.0	58	70	363 434	434.8	3557	62.1	82.12
Tdic1B	849.7	65	39.1	45	64	1 181 661	700.4	1092	77.8	79.46
Tdic2A	804.7	92	36.9	43	54	327 364	288.3	2345	31.9	69.68
Tdic2B	708.1	93	34.8	39	74	350 258	477.1	4266	50.1	78.88
Tdic3A	647.7	93	35.5	44	70	427 274	431.1	2787	50.2	79.90
Tdic3B	742.1	92	38.0	40	70	360 242	552.9	4707	55.3	79.93
Tdic4A	864.0	76	28.9	41	54	638 342	388.6	1114	41.1	70.52
Tdic4B	893.6	87	30.3	40	74	592 030	519.0	2479	64.9	83.22
Tdic5A	659.8	98	33.8	51	58	455 525	353.1	1913	41.3	72.93
Tdic5B	842.0	95	34.1	41	62	572 876	441.2	1927	50.5	76.24
Tdic6A	958.4	97	34.0	53	80	252 227	392.7	4938	54.4	82.09
Tdic6B	764.1	53	34.3	42	70	905 302	624.3	1522	67.0	79.69
Tdic7A	813.5	59	34.5	47	60	649 826	497.4	1822	51.1	73.85
Tdic7B	772.9	75	30.0	39	70	730 770	585.1	2090	60.3	79.72

a Chromosome sizes were assumed to be similar to the estimated sizes for *T. turgidum* cv. Timilia (Venora *et al*., 2002), calculated by dividing 1C genome size by relative length.

### *T. dicoccoides* genome is swamped by repetitive elements

**Figure 1:**
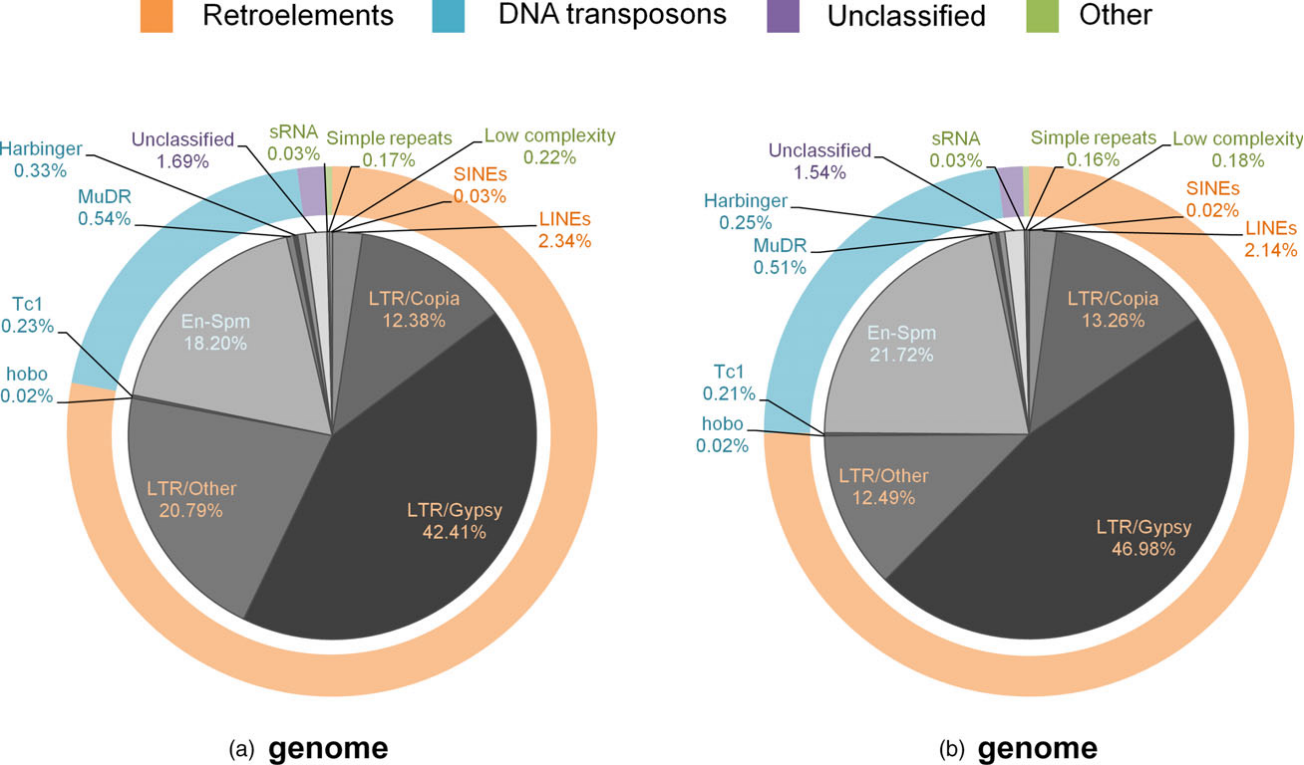
Distribution of repetitive elements in the A and B subgenomes. Repeat annotations of three chromosomes belonging to each subgenome were combined to assess the repetitive landscape of the subgenomes. DNA: DNA transposons; En‐SPM/CACTA, Harbinger, MuDR/Mutator, Tc1/Mariner and hobo/Activator are DNA transposon subfamilies, while long terminal repeats (LTR) elements and long and short interspersed elements (LINEs, SINEs) are retroelements.

As a hallmark of *Triticeae* genomes, chromosome assemblies were also swamped by repetitive elements which indicated an overall repeat content of 78.2% for the entire genome, marginally underestimated due to the exclusion of ultra‐short contigs. While Class I retroelements dominated the overall repetitive landscape, at the family level, the Jorge family of Class II DNA transposons (En‐Spm superfamily) was prominent, reaching as many as 15% of all bases masked as repetitive elements in all chromosome assemblies. Among repeat families, the most striking difference was observed for undetermined LTR elements, which collectively comprised over one‐fifth of all repeat annotations from A‐genome chromosomes, in contrast to B‐genome chromosomes, where these elements comprised only ~12.5% of all repeat annotations combined (Figure 1).

### *T. dicoccoides* genome is highly populated with genes that are conserved among grasses

To identify genes encoded by the *T. dicoccoides* chromosomes and explore syntenic relationships, chromosome assemblies were compared against the fully annotated proteomes of the related grasses *Brachypodium*, rice and sorghum and high‐confidence proteins of its close relative, barley, in addition to transcript assemblies and predicted proteins of *T. dicoccoides* (Akpinar *et al*., 2015a; Avni *et al*., 2017) (Table S1, Figure S1). To avoid redundancy in gene number estimations due to orthologous relationships, *Brachypodium*, rice, sorghum and barley genes were clustered into nonredundant orthologous groups based on sequence similarity using OrthoMCL (Li *et al*., 2003). In total, 60 392 contigs were associated with 14 882 nonredundant orthologous genes from related grasses, representing orthologous coding loci. Of these, loci found on only one chromosome, on homeologous group chromosomes or all chromosomes, were excluded. Remaining 6430 coding loci found on two or more chromosomes corresponding to the same orthologous grass gene may point to putative interchromosomal gene duplication or gene loss events. Overall, assuming an average coding sequence length of 2772 bases for wheat (Luo *et al*., 2013), these observations suggested that the entire *T. dicoccoides* genome may encode ~37 000 conserved genes that have orthologs in the four related grass genomes, with a peak of conserved gene fraction of 1.3% for chromosome Tdic4A. The estimation of total gene content of the genome should take functional intra‐chromosomal gene duplicates that widely diverged from their grass orthologs (neo‐functionalization) and genes that are specific to the wheat lineage into account, while putting special care on the well‐known prevalence of nonfunctional pseudogenes in wheat genomes (Choulet *et al*., 2010).

**Figure 2:**
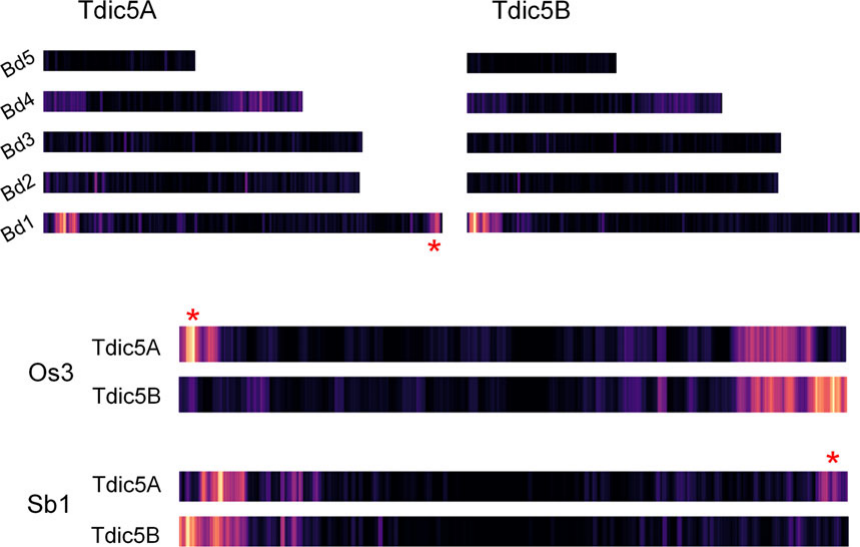
Syntenic relationships of homeologous group 5 chromosomes with the *Brachypodium* genome, rice chromosome 3 (Os3) and sorghum chromosome 1 (Sb1). For simplicity, only chromosomes of interest are shown for rice and sorghum. Chromosome lengths for *Brachypodium* are relative to each other, while for rice and sorghum, chromosome lengths are arbitrary. Red asterisks highlight syntenic regions observed for Tdic5A but not Tdic5B.

Due to their shared ancestry, cereal genomes exhibit widespread colinearity, forming large ‘syntenic’ regions on chromosomes that carry orthologous genes (The International Brachypodium Initiative, 2010). Comparison of the chromosome assemblies to the related grasses reproduced the well‐known patterns of synteny at the chromosome level, providing evidence for the well‐documented 4AL‐5AL‐7BS translocation, as well (Figure S2). While Tdic5A and Tdic5B had similar conservation patterns in general, a unique region of synteny to the distal regions of Bd1 (Bd1 for *Brachypodium distachyon* chromosome 1) and Sb1 (Sb1 for *Sorghum bicolor* chromosome 1) and to the proximal region of Os3 (Os3 for *Oryza sativa* chromosome 3), was observed only for Tdic5A (Figure 2).

### Overview of the structural comparison of wild and modern wheat genomes

To explore the overall conservation between modern and wild wheat genomes, chromosome assemblies were compared against the genomes of wild emmer wheat Zavitan (Avni *et al*., 2017) and bread wheat cultivar Chinese Spring (IWGSC RefSeqv1). We individually mapped the chromosome assemblies to each genome and used any sequence mapping to specific locations on both the Zavitan and Chinese Spring genomes as links to connect the two respective positions. For each of the 14 chromosomes, approximately 400 000–500 000 such links demonstrated large‐scale structural variations between these wild and domesticated genotypes. As expected from the close evolutionary relationship between these two species, extensive colinearity between orthologous chromosomes was evident (Figure S3). Despite the extensive conservation between the closely related tetraploid wild genomes, we identified over 45M sequence variations, including single nucleotide polymorphisms (SNPs) and InDels, between our chromosome sequences and the Zavitan genome that passed stringent filtering criteria (Akpinar *et al*., 2016) (Table S2). These SNPs could be useful in designing novel SNP markers to distinguish regions of interest within these highly similar genomes.

### MiRNAs from the noncoding RNA pool hint at potential toolboxes of *T. dicoccoides* in response to stress

Chromosome assemblies contained 28 011 putative precursor loci for miRNA biogenesis, representing 59 miRNA families (Table S3). Of these, expression evidence was present for 49 families (83%) at the small RNA (sRNA) and/or pre‐miRNA level, among previously published durum wheat sRNA sequences and *T. dicoccoides* transcripts (Akpinar *et al*., 2015a; Distelfeld, 2016; Liu *et al*., 2015). Predicted miRNAs with precursor sequences containing transposable elements (TEs) by more than 50% of their lengths were classified as ‘low confidence (LC)’, as TE‐associated miRNAs are not easily distinguished from siRNAs, particularly from genome sequence data alone. The precursor sequences of these LC miRNA families predominantly contained Class I DNA transposons, abundantly from the Mariner superfamily (Table S3). Notably, 11 miRNA families were processed from both LC precursors and precursors that contained few to no TEs (classified as ‘high confidence (HC)’). Seventeen miRNA families contained loci on all chromosomes, suggesting functional redundancy and/or potential TE‐mediated proliferation events and loci for 49 miRNA families were common to both subgenomes (Figure S4). On a broader scale, using the same identification procedure, precursor sequences for 26 HC miRNA families identified from chromosome assemblies were also present in the whole‐genome sequences of *T. dicoccoides* genotype Zavitan (Avni *et al*., 2017) (27 HC miRNA families predicted in total) and the hexaploid *T. aestivum* cv. Chinese Spring (31 HC miRNA families predicted in total), suggesting a core collection of regulatory miRNAs in polyploid wheats. In all three species, the total number of miRNA encoding loci was the highest in the B subgenomes. When mapped back to the genome, majority of mature miRNAs predicted from chromosome assemblies aligned with intergenic regions (94.35% of all predicted mature miRNAs), pointing out to a vast genomic source for miRNA biogenesis. On average, only 0.11% and 5.54% of predicted miRNAs aligned to exons and introns, respectively, of gene models (IWGSC RefSeqv1), indicating potential autoregulatory circuits.

Functional annotation of the putative targets of *T. dicoccoides* miRNAs identified from the chromosome assemblies revealed an array of biological processes, molecular functions and cellular components, with subtle differences across subgenomes and homeologous groups. These potential miRNA‐target pairs hint at an intriguing toolbox that can be used by *T. dicoccoides* to cope with stress conditions, including nuances for fine‐tuning. For instance, *Tsn1*, a biotic stress‐related gene involved in the response to tan spot disease in durum wheat, was a target of the miR2118 family members from multiple chromosomes with slightly different mature miRNA sequences (Table S3). Whole‐genome network of potential miRNA‐target pairs can be of substantial importance to understand the regulation of a given phenotype with its full dimensions.

In addition to miRNAs, precursors for two other classes of noncoding RNAs, tRNAs and long noncoding RNAs (lncRNAs) were identified from the chromosome assemblies. Consistent with previous observations, a marked abundance of tRNA^Lys^ genes, speculatively resulting from co‐proliferation following an ancient TE capture, was observed for all chromosomes (Figure S5) (Akpinar *et al*., 2014, 2015b; Lucas *et al*., 2014; Tanaka *et al*., 2013). As lncRNAs are typically mined from transcriptomics data, lncRNA candidates on chromosome assemblies were explored using a comparative approach. Potential lncRNA sequences were extracted from a combined set of RNA‐sequencing data from *Triticum turgidum* ssp. *dicoccoides* genotype Zavitan, six different tissues of *T. aestivum*, and also drought stressed and irrigated samples of *T. aestivum* with a strict set of elimination steps (Cagirici *et al*., 2017) (Experimental Procedures). A total of 89,623 lncRNAs passing all elimination steps were then mapped to the *T. dicoccoides* chromosome assemblies and genome sequences of wild emmer wheat Zavitan (Avni *et al*., 2017) and bread wheat cultivar Chinese Spring (IWGSC RefSeqv1) to confidently identify a core set of lncRNAs. Importantly, of 83 822 lncRNAs common to all three genomic resources, 23 713 were potential targets of mature miRNAs from chromosome assemblies (Table S4). These miRNA‐lncRNA associations may provoke target mimicry, within stress response pathways as well. For instance, a member of the miR437 family (mature miRNA sequence: AAAGUUAGAGAAGUUUGACUU) targeted an aquaporin *PIP1‐5* transcript in *T. dicoccoides*, along with by 8, 24 and 89 lncRNAs in identified from leaf, seed and root tissue transcriptomes of *T. aestivum*, respectively. It is tempting to speculate that under drought stress, target mimicry imposed by lncRNAs can alter miRNA function, resulting in reduced down‐regulation of aquaporin proteins and improved water uptake.

### *T. dicoccoides* chromosome assemblies cover several agronomically important loci

Chromosome assemblies contained several genomic loci coding for agronomically relevant traits, which not only demonstrated the utility of the assemblies but, also in some cases, provided novel insights (Data S1). Domestication is a key event shaping the wheat genome. In barley, *nonbrittle rachis 1* (*Btr1*) and *nonbrittle rachis 2* (*Btr2*) control an important domestication‐related trait, ‘Brittle Rachis (BR)’. Loss‐of‐function mutations in either of these genes disrupt the BR phenotype that enables free dispersal of grains at maturity (Pourkheirandish *et al*., 2015). In tetraploid wheat, homeologous copies of both *Btr1* and *Btr2* genes were found on chromosomes 3A and 3B (Avni *et al*., 2017). In this study, two contigs, Tdic3A‐contig10048876 and Tdic3B‐contig9087392, contained coding regions for *Btr1‐A* and *Btr2‐B* (designated as *TdicBtr1‐A* and *TdicBtr2‐B*), respectively, as indicated by their sequence similarities to barley *Btr* genes. Notably, *TdicBtr1‐A*, with 93% sequence identity to the wild barley (*Hordeum vulgare* subsp. *spontaneum*) *Btr1* gene (KR813340.1), had a 2‐bp deletion at position 290 that resulted in a premature stop codon. Therefore, the predicted translated product of *TdicBtr1‐A* was a 97‐amino acid peptide that was likely nonfunctional. By contrast, *TdicBtr2‐B* was 88% identical to its wild barley ortholog (KR813339.1) and was translated into a full‐length 198 amino acid protein product (Table 2).

**Table 2:** BTR protein characteristics in wild tetraploid and modern hexaploid wheat

	Wild emmer wheat ‘Zavitan’	*T. dicoccoides* chromosome assemblies (This study)	*T. aestivum* Chinese Spring (IWGSC RefSeq)
BTR1‐A	196 residues	97 residues	97 residues
	Functional	Nonfunctional	Nonfunctional
BTR1‐B	196 residues	196 residues functional	196 residues
	Functional		Nonfunctional
BTR2‐A	198 residues	173 residues	198 residues
	Functional	Nonfunctional	Functional
BTR2‐B	198 residues	198 residues	198 residues
	Functional	Polymorphic	Functional

A closer look at the protein level revealed candidate sequences on two contigs on chromosome 3A and three contigs on chromosome 3B for *Btr2‐A* and *Btr1‐B*, respectively. Potential coding regions on contigs from chromosome 3B shared relatively low sequence similarity with *Btr1* sequences, which is consistent with the presence of Btr‐like genes along the genome (Pourkheirandish *et al*., 2015). However, Tdic3A‐Cluster30852 contained 494 bases from the 3ʹ end of a potential *Btr2‐A* gene separated from its 61‐base 5′ region by a 3.7‐kb unrelated sequence. Together, these segments of the *Btr2‐A* gene shared high sequence identity with its barley ortholog (90%), and its sequence was identical to that of Zavitan *TtBtr2‐A* (Avni *et al*., 2017). In addition, the unrelated sequence separating the 3ʹ and 5ʹ ends of *Btr2‐A* carried TE elements including both DNA transposons and retroelements. This organization suggested that a TE insertion within the coding sequence of *TdicBtr2‐A* could have relocated the first 61 bases at the 5ʹ end to a downstream position, splitting this sequence into two segments and resulting in the loss of a genomic sequence encoding 15 amino acids in the translated product. Notably, Cluster30852 originated from the merging of two Tdic3A contigs that mapped to the Zavitan 3A chromosome at positions 41 bases apart. Such an organization could have been the result of ambiguous (and erroneous) mapping due to the presence of repetitive sequences, although the presence of flanking nonrepetitive sequences would reduce this possibility. This observation could also be explained by a small‐scale genome rearrangement on Tdic3A in our assembly with respect to the Zavitan genotype. In this case, even though the 5ʹ and 3ʹ fragments were identical to the BTR2 protein, such an organization should abolish its function completely (Table 2, Figure S6).

The chromosome assemblies also covered two vernalization genes that are important regulators of flowering in wheat. Tdic5A‐contig17747970 and Tdic5B‐contig15503489 contained homeologous coding regions for *Vrn1*, while Tdic7A‐contig20604678 potentially contained coding sequences for *Vrn3*. Intriguingly, two contigs from Tdic4B might harbour coding sequences for the *Vrn2* locus, along with TE‐related sequences. The *Vrn2* locus, which was mapped to the 5A chromosome, was organized as two tandemly duplicated genes, *ZCCT1* and *ZCCT2*. The protein products of both genes were composed of a putative zinc finger domain and a CCT domain, and they shared 76% sequence identity (Distelfeld *et al*., 2009). The 11‐kb Tdic4B‐contig10186201 contained a two‐exon ORF that was translated into a 217‐residue peptide. This translated product shared 93% similarity with *T. turgidum* ZCCT2‐B2a (ACI00359.1). Importantly, the product encoded by contig10186201 did not exhibit any of the three mutations at positions 16, 35 and 39 of the CCT domain that are predicted to disrupt protein function (Distelfeld *et al*., 2009). The translation product of a second contig on Tdic4B, contig10211027, consisted of 209 amino acids and was 97% similar to *T. turgidum* ZCCT1‐B1 (ACI00355.1). Both of these ORFs were surrounded by TEs, including the highly promiscuous Gypsy family of retrotransposons. Whether these ORFs represent a fully functional *Vrn2*‐like locus that was translocated into Tdic4B awaits further study.

Leaf rust caused by *Puccinia triticina* is an important disease that affects wheat production worldwide(Sela *et al*., 2012; Xie and Nevo, 2008). A leaf rust resistance gene, *Lr10*, was located inside the 13‐kb contig6213438 from Tdic1A, which may potentially represent a new allele (Data S1). The *Lr10* locus resided within in a gene‐rich segment on the distal end in close proximity to another resistance gene, *Pm3*, conferring resistance against powdery mildew disease. In our chromosome assemblies, the 17‐kb contig, Tdic1A‐contig6388763, carried a coding region for the *Pm3* locus. Importantly, a number of resistance genes against Hessian fly have also been mapped to the same gene‐rich region on 1AS, a few of which have already been introgressed from close and distant relatives into hexaploid wheat (Data S1).

Autophagy is a process in which cellular contents are degraded, resulting in either recycling of cytoplasmic components or the elimination of damaged or toxic molecules inside the cell. This process plays an important role in both abiotic and biotic stress responses (Klionsky *et al*., 2016). For instance, wheat homeologs of ATG6 play a role in responses to fungal pathogens in addition to abiotic stress factors (Yue *et al*., 2015). Additionally, the autophagy‐related gene, *TdATG*8, which was recently cloned from wild emmer wheat, is responsive to water deficit (Kuzuoglu‐Ozturk *et al*., 2012). The *T. dicoccoides* chromosome assemblies obtained in this study covered several autophagy‐related genes, including previously cloned *ATG4*, *ATG8* and *ATG6* (Nagy *et al*., 2014; Pei *et al*., 2014; Yue *et al*., 2015). Considering the importance of autophagy‐related genes in plant metabolism and stress responses which can be modulated for the improvement, all autophagy genes were mined and comparatively analysed in tetraploid Zavitan and hexaploid Chinese Spring genomes. In total, 44 coding regions corresponding to highly conserved 18 isoforms of ATG genes were identified across all wheat chromosomes (Data S2). *Atg8* isoforms were found in three separate locations on homeologous groups 2 and 5 chromosomes, consistent with previous findings (Kuzuoglu‐Ozturk *et al*., 2012). ATG genes were also conserved at the protein level in close relatives rice and *Brachypodium*, as well as in *Arabidopsis thaliana* albeit to a lesser extent (Table S5).

**Figure 3:**
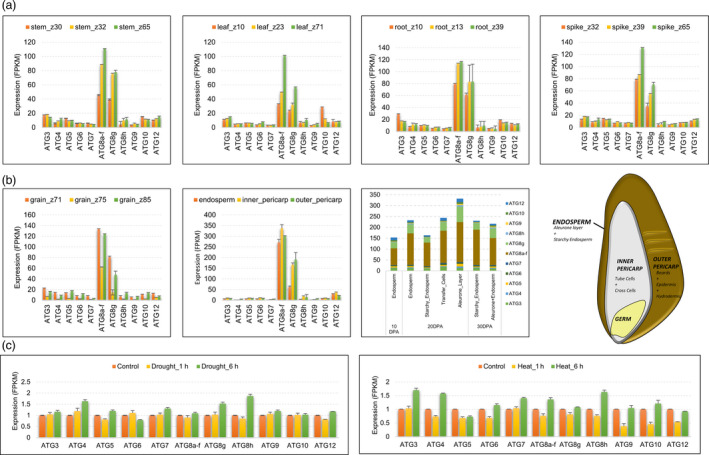
Expression pattern of Autophagy‐related genes from hexaploid wheat. (a) Tissue and time‐zone specific expression of ATG genes. (b) The expression pattern of ATG genes in grain tissue and grain tissue layers. (c) The ATG expression pattern under heat and drought stresses. The expression is normalized to control.

To gain deeper insight into the functional roles of ATG genes, we assessed the differential expression patterns of these genes including stress responsive pathways and cis regulatory elements. The promoter regions of ATG genes hosted recognition sites for many different transcription factor (TF) families such as WRKY, GATA, TCR and ERF, suggesting a complex interplay of multiple molecular pathways (Figure S7). Importantly, recognition sites for AP2, B3, TCP, NAC‐NAM, bZIP, NF‐YB TF families were detected in the promoter regions of all ATG genes, which indicates autophagy is commonly recruited in an array of developmental and stress response‐related processes, under different genetic and/or environmental cues. Consistent with these scheme, ATG genes were differentially expressed in different tissues, developmental stages and environmental conditions (Figure 3). A hallmark of autophagy, Atg8 expression, was prominent in all tissues tested, potentially indicating a basal level of autophagy as a normal developmental process (Figure 3a). Furthermore, ATG genes were also differentially expressed in different layers of grain, with a relatively lower expression profile in endosperm compared to inner and outer pericarp tissues. ATG expression appeared to be high in transfer cells in grain, pointing out an increased autophagic activity in these cells (Figure 3b). Finally, ATG genes were also responsive to abiotic stress factors, such as drought and heat (Figure 3c), together with developmental changes such as senescence and photomorphogenesis.

## Discussion

Due to its high agronomic importance, intensive efforts have focused on achieving whole‐genome sequencing in wheat, which would open new possibilities for molecular breeding programs (Berkman *et al*., 2012a; Feuillet *et al*., 2011). Early efforts included large‐scale sequencing of individual chromosomes (Berkman *et al*., 2011, 2012b; Lucas *et al*., 2014; Vitulo *et al*., 2011). More recently, a chromosome‐based draft genome sequence was released (Mayer *et al*., 2014), followed by an improved genome assembly (Zimin *et al*., 2017), and the first reference‐quality sequence of the largest wheat chromosome 3B was published (Choulet *et al*., 2014). To fully exploit genomics‐based wheat breeding research, the next objective will be to unlock the genomes of related germplasm. Wild emmer wheat, *Triticum turgidum* ssp. *dicoccoides*, is the wild relative of the tetraploid durum wheat progenitor *Triticum turgidum* and exhibits a remarkable level of adaptation, thereby serving as a valuable source of genetic diversity. Currently available resources for large‐scale genomics analyses in *T. dicoccoides* include sequencing of its 5B chromosome(Akpinar *et al*., 2015b), and the recently published genome assembly (Avni *et al*., 2017). In the current study, we expand these resources by high‐throughput sequencing and assembly of all 14 chromosomes of *T. dicoccoides* (collectively referred as chromosome assemblies, hereafter).

*Triticeae* genomes are notorious for their high DNA repeat content, reaching 80%–90% of the entire genome. Consistent with previous observations, our chromosome assemblies contained a large fraction of repetitive elements. The relative abundance of undetermined LTR elements, differing widely between subgenomes, supports the view that relatively few LTR elements likely existed in the B‐genome progenitor, resulting in the accumulation of more recent, and therefore more ‘classifiable’, transposon‐mediated amplifications in the modern B‐genomes (Mayer *et al*., 2014). The nonrepetitive fraction of the chromosome assemblies reproduced the well‐known syntenic relationships between related grasses, *Brachypodium*, rice and sorghum, including the 4AL‐5AL‐7BS translocation (Hernandez *et al*., 2012; Kantar *et al*., 2012) (Figure 2). This observation provides additional evidence at the sequence level that this translocation event dates back to the differentiation of the *T. turgidum* subspecies, possibly before the emergence of *T. dicoccoides* (Hao *et al*., 2013). In total, 74,653 contigs exhibited significant sequence similarity to *T. dicoccoides* transcripts and proteins, but not to any related grass genes. However, consistent with the prevalence of pseudogenes and gene fragments in the wheat genome (Choulet *et al*., 2010), only 30% of these contigs covered more than 70% of the target wheat gene, a subset of which should include genes specific to the wheat lineage,

Overall, 59 miRNA families, including five that have not been previously reported (miR158, miR2109, miR414, miR5825 and miR6026), were predicted to be encoded by the entire *T. dicoccoides* genome, using a homology‐based pipeline (Alptekin *et al*., 2017). A subset of precursor sequences were highly associated with repetitive elements, in particular Class II DNA transposons, supporting the view that DNA transposons may be involved in miRNA biogenesis and evolution (Akpinar and Budak, 2016; Li *et al*., 2011b). Predicted targets of miRNAs, including mRNA and lncRNA transcripts, hinted to the post‐transcriptional regulatory pathways in wheat. For instance, of the novel families reported, miR2109, identified only in Tdic3A, has been reported in legumes in NBS‐LRR‐mediated defence signalling (Zhai *et al*., 2011). This miRNA family targets an ATP‐dependent DNA helicase (Table S3). Although the underlying molecular mechanism remains unknown, the overexpression of a pea DNA helicase increases salinity tolerance in tobacco and rice (Sahoo *et al*., 2012; Tuteja, 2010). In addition, several miRNAs associated with biotic stress responses only in dicot species to date were detected in chromosome assemblies, such as the miR482 family implicated in responses to fungal infection in cotton (Zhu *et al*., 2013). Like miR482, miR6026, which was previously identified only in tomato and potato, is associated with innate immune receptors (Li *et al*., 2011a). Comparative analysis of miRNAs indicated that several miRNA families are conserved across tetraploid and hexaploid wheat genotypes (Figure S4).

Chromosome assemblies cover several agronomically important loci demonstrating their utility. These assemblies, most of which are a few kilobases long, also include upstream and downstream sequences for these loci, which can be valuable for cloning genes of interest (as in the case of the *QHf.osu‐1A* QTL for Hessian fly resistance), discovering new alleles, elucidating the genetic basis of related traits including regulatory regions and developing new molecular markers that can be utilized in breeding programs. For instance, while a recent study failed to identify the 1B allele of *MTP1* in hexaploid wheat (Vatansever *et al*., 2017), potential *MTP1* homologs were identified in two Tdic1B contigs, each encoding a protein with an intact cation efflux domain characteristic of MTP proteins and with high sequence similarity to known MTP1 proteins (Data S1). Intriguingly, sequence similarity searches revealed a potential coding region for the *Vrn2* locus on Tdic4B, which could have resulted from TE capture‐based translocation, although this locus was originally mapped to the 5A chromosome (Yan, 2004). In fact, additional duplication events involving the *Vrn2* locus might have occurred (Distelfeld *et al*., 2009). Further functional studies should clarify whether this locus on Tdic4B is transcribed and translated into a fully functional protein, thereby representing new alleles for the vernalization response in wheat.

A coding region on Tdic3A assembly was identified as the *Btr1‐A* allele for the domestication‐related BR phenotype, which is translated into a peptide identical to the wild emmer genotype Zavitan protein BTR1‐A. Intriguingly, our Tdic3A assembly indicated that a second region, candidate *Btr2‐A* coding region, contains an extensive rearrangement by TE insertion that likely disrupts the function of the translated peptide (Figure S6). While we could not accurately identify the coding region for *Btr1‐B* on Tdic3B, where candidate regions resemble *Btr1‐like* sequences known to exist in the genome, the *Btr2‐B* gene on Tdic3B was capable of being translated into the full‐length 198‐residue protein. Interestingly, this protein is only 91% identical to the BTR2‐B protein in wild Zavitan (Avni *et al*., 2017). Interestingly, BTR2 protein sequences were identical in the wild tetraploid Zavitan and domesticated hexaploid Chinese Spring (Table 2). Although loss‐of‐function mutations in the two *Btr1* genes was previously reported to be required for the nonBR phenotype of the domesticated varieties in tetraploid wheat (Avni *et al*., 2017), the BR phenotype may be under a more complicated regulation, given that our chromosome assemblies suggested a nonfunctional copy of the *Btr1A* in another *T. dicoccoides* accession that is identical to the domesticated hexaploid Chinese Spring copy, which otherwise carries all functional copies (Table 2) and also identical to the domesticated tetraploid genotypes (Avni *et al*., 2017). Indeed, we found an intact Q‐locus on Tdic5A‐contig17630980. The Q‐locus is known to have pleiotropic effects on free threshing, rachis fragility and spike shape. Therefore, it is tempting to speculate that in some wild genotypes, mutations in the BR locus that would otherwise disrupt the BR phenotype are ‘rescued’ by other loci, particularly those that exhibit pleiotropy.

The expression of autophagy‐related genes, also covered by our chromosome assemblies, indicated differential regulation of autophagy in tissue, developmental stage and environmental condition‐dependent backgrounds. Compartmentalization of nutrients from source to sink and the differential regulation of ATG genes in the different layers of grain tissue proved insights about this role of autophagy in wheat (Figure 3). The higher expression of ATG genes particularly in aleurone layer and transfer cells may suggest high autophagic activity in these cells which contributes to movement of nutrient to endosperm for long‐term storage. In‐depth elucidation of the role of autophagy in nutrient compartmentalization may help to increase the nutritional quality of both bread and durum wheat.

Finally, despite extensive colinearity of their genomes, over 45M SNPs and short InDels were discovered between our chromosome assemblies, wild emmer Zavitan and the bread wheat Chinese Spring, which can be utilized novel molecular markers. In addition, 21 contigs from the chromosome assemblies, which were analysed in detail for the selected traits described above, were used to identify simple sequence repeats (SSRs) and TE junctions within or in the vicinity of coding regions. In total, 41 SSRs and 182 TE junctions were identified for potential use as SSR‐based and insertion site‐based polymorphism (ISBP) markers (Paux *et al*., 2010), demonstrating the utility of these chromosome assemblies in assisting wheat improvement efforts (Table S6). Taken together, these sequence variations and the genome‐wide sequence data can be used to expand and saturate map‐based resources for tetraploid wheat to support breeding programs.

## Experimental procedures

### Chromosome sorting, sequencing, assembly and filtering

Bivariate flow karyotyping and chromosome sorting were carried out as previously described (Akpinar *et al*., 2015b) using *Triticum dicoccoides* (2*n* = 4*x* = 28), genotypes TR191 (Karacadag region of Turkey) and MvGB436 (provided by Dr. István Molnár, Centre for Agricultural Research, Hungarian Academy of Sciences, Martonvásár, Hungary). Only chromosomes 2A and 4A were derived from genotype MvGB436, as flow karyogram did not allow separation of these chromosomes to an acceptable purity in the other genotype. Chromosomal DNA was amplified by isothermal multiple displacement amplification (MDA) (Data S2).

Sequencing of amplified chromosomal DNA was carried out by Centrillion Genomic Services, Centrillion Technologies (Palo Alto, CA, http://www.centrilliontech.com/) on high output mode HiSeq 2500 v3 at a read length of 2x100PE. The quality metrics of the raw reads were assessed using FASTQC software (http://www.bioinformatics.babraham.ac.uk/projects/fastqc/). The Illumina paired‐end reads were *de novo* assembled using the ABySS tool (Simpson *et al*., 2009). The k‐values for the sequence assemblies were determined using KmerGenie (Chikhi and Medvedev, 2014) (Table 1). ABySS scaffolds were then mapped to the chromosome assemblies of *T. dicoccoides* genotype Zavitan (Avni *et al*., 2017). Scaffolds mapping to the respective Zavitan chromosomes at a distance closer than 50 nucleotides were merged with ‘N's using custom Python scripts, thereby producing the final reference‐guided assemblies. Repetitive elements were identified by comparing the chromosome assemblies against the MIPS Repeat Element Database v9.3 p for Poaceae (ftp://ftpmips.helmholtz-muenchen.de/plants/REdat/) using RepeatMasker v.3.3.0 software (http://www.repeatmasker.org/) (Nussbaumer *et al*., 2013).

### Protein‐coding genes and syntenic relationships

Protein‐coding genes were identified through BLAST searches against the fully annotated proteomes of *Brachypodium distachyon* (v1.2, http://mips.helmholtz-muenchen.de/plant/brachypodium) (The International Brachypodium Initiative, 2010), *Oryza sativa* (IRGSP‐1.0, http://rapdb.dna.affrc.go.jp/download/irgsp1.html) (Tanaka *et al*., 2008), *Sorghum bicolor* (v1.4, http://mips.helmholtz-muenchen.de/plant/sorghum/) (Paterson *et al*., 2009) (1E‐6, ‐length 30, ‐ppos 75); high‐confidence *Hordeum vulgare* proteins (http://mips.helmholtz-muenchen.de/plant/barley/) (Mayer *et al*., 2012) (1E‐6, ‐length 30, ‐ppos 90) and *Triticum dicoccoides* transcripts assembled from RNA‐sequencing data (Akpinar *et al*., 2015a; Avni *et al*., 2017) and predicted proteins (Avni *et al*., 2017) (1E‐30, ‐length 100, ‐pident 99 for transcripts; 1E‐6, ‐length 30, ‐ppos 99 for proteins). A ‘Best Reciprocal Hit’ approach was adopted, and only the best reciprocal hits in blastx and tblastn searches with the given parameters were accepted as significant matches.

To estimate gene number, a nonredundant list of orthologous grass genes was constructed from the *B. distachyon*, *O. sativa*, *S. bicolor* and *H. vulgare* proteins described above using the OrthoMCL pipeline (http://www.orthomcl.org/orthomcl/) (Li *et al*., 2003) (Data S2).

Syntenic relationships were visualized as circle plots and heatmaps generated using Circos software (Krzywinski *et al*., 2009) and in‐house Python scripts. Ribbons on the circle plots were obtained by bundling >200 links along 1‐Mb intervals. Gene densities for *B. distachyon*, *O. sativa* and *S. bicolor* were counted on 500‐kb intervals (light blue and light grey). The genomic positions of annotated genes were obtained from the MIPS database of plants (http://mips.helmholtz-muenchen.de/plant/genomes.jsp). For all BLAST searches, BLAST+ stand‐alone toolkit, version 2.2.31 was used (Camacho *et al*., 2009). All computational analyses were carried out on a High Performance Computing Cluster. All functional annotations were performed using BLAST2GO (Conesa and Götz, 2008); the initial BLAST steps were run locally against all *Viridiplantae* proteins (1E‐6, ‐outfmt 5, ‐max_target_seq 1).

### Putative noncoding RNA species

A homology‐based miRNA prediction approach was utilized to annotate miRNA‐encoding sequences as described previously, with slight modifications (Alptekin *et al*., 2017; Kurtoglu *et al*., 2013, 2014; Lucas and Budak, 2012) (Data S2). Expression analysis of the predicted miRNAs was performed at both the pre‐miRNA and mature miRNA level, as described in Data S2. Target sequences for putative miRNAs were predicted with the web‐tool psRNATarget (http://plantgrn.noble.org/psRNATarget/), using transcripts assembled from RNA‐sequencing data from two *T. dicoccoides* genotypes (Akpinar *et al*., 2015a; Cagirici *et al*., 2017), along with the *T. dicoccoides* whole‐transcriptome assembly (Avni *et al*., 2017). For comparative analyses, miRNA identification was carried out on the genome assembly of wild emmer genotype Zavitan (Avni *et al*., 2017) and hexaploid wheat cv. Chinese Spring (RefSeqv1, International Wheat Genome Sequencing Consortium, https://urgi.versailles.inra.fr) using the same approach and target transcripts were identified among whole‐transcriptome assembly (Avni *et al*., 2017) for Zavitan and high‐confidence coding sequences (ftp://ftpmips.helmholtz-muenchen.de/plants/wheat/IWGSC/) for Chinese Spring. Putative tRNA genes were identified using the tRNAscan‐SE 1.21 program(Lowe and Eddy, 1997) with default parameters for eukaryotic genomes. Pseudogenes and other undetermined annotations were excluded.

Identification of lncRNAs was performed using three available transcriptome data sets from (i) wild emmer genotype ‘Zavitan’ (both high‐confidence and low‐confidence transcripts, (https://wheat.pw.usda.gov/graingenes_downloads/Zavitan/), (ii) hexaploid wheat Chinese Spring (http://www.earlham.ac.uk/organisms/wheat) and (iii) Drought‐stressed and irrigated samples of Chinese Spring. The coding potentials of these transcriptome sequences were assessed with CNCI (https://github.com/www-bioinfo-org/CNCI) (Sun *et al*., 2013), and ORFs were identified using Transdecoder (https://transdecoder.github.io/). Transcripts with protein‐coding potential and/or with potential ORFs encoding polypeptides >100 amino acids in length were discarded. Remaining transcripts were compared against known protein sequences of *B. distachyon*, *O. sativa*, *S. bicolor* and *H. vulgare*, as above, and against protein sequences of *Triticum aestivum* (UniProt) and *Triticum turgidum* (NCBI, retrieved from 7 February 2017), together with reviewed protein sequences from the Swiss‐Prot database with both ‘blastx’ (1E‐5, ‐length 30, ‐ppos 80) and ‘tblastn’ (1E‐5, ‐length 30, ‐pident 80). BLAST searches were also performed against Unigene sequences of *B. distachyon* (build #2), *O. sativa* (build #86), *S. bicolor* (build #30), *H. vulgare* (build #59) and *T. aestivum* (build #63) and EST sequences of *T. turgidum* (NCBI, retrieved from 7 February 2017) (1E‐30,‐length 90, ‐identity 80). In addition, BLAST searches were performed against the organellar genomes of *T. aestivum* (gene bank ID: NC_007579.1 and NC_002762.1) and *T. turgidum* (KJ614397.1), together with noncoding RNA sequences (rRNA, tRNA, snRNA and snRNA). Transcript sequences showing any homology in these analyses were discarded and candidates longer than 200 nucleotides were accepted as putative lncRNAs. The lncRNAs were mapped onto the genome assemblies of wild emmer genotype Zavitan and Chinese Spring, as well as the *T. dicoccoides* chromosome assemblies, using GMAP (‐cross‐species, ‐f ‘2) (http://research-pub.gene.com/gmap/) (Wu and Watanabe, 2005). For positive alignments, only lncRNAs mapping through their entire lengths with an alignment score of at least 40 were accepted. Finally, the lncRNA transcripts that contain noncanonical splice sites were checked in depth to assess whether these transcripts may still have potential coding features that eluded detection simply due to strandedness. This final elimination discarded the lncRNAs with noncanonical splice sites if their reverse complemented sequences coded for canonical transcripts.

### Identification of SSRs and TE junctions and SNPs

SSRs from chromosome assembly contigs fully covering selected genes from *T. dicoccoides* and *T. turgidum* were identified independently using the Gramene SSR tool (http://archive.gramene.org/db/markers/ssrtool) and the MISA‐MIcroSAtellite identification tool (http://pgrc.ipk-gatersleben.de/misa/). TE junctions for which ISBP markers can be designed were identified using IsbpFinder (Paux *et al*., 2010). SNPs were identified as previously described (Akpinar *et al*., 2016).

## Data availability

The data sets generated during the current study are available in the [TdicA‐B] repository, under the accession numbers PRJEB24100 (raw reads) and PRJEB25714 (chromosome assemblies). The data sets generated and/or analysed during the current study, including custom scripts, are also available from the corresponding author on request.

## Competing interests

The authors declare no competing financial interests.

## Supporting information


**Figure S1** Sequences from chromosome assemblies associated with conserved and non‐conserved genes.

**Figure S2** Syntenic relationships between *T. dicoccoides* and related grasses *Brachypodium* (Bd), rice (Os) and sorghum (Sb).

**Figure S3** Overview of the structural genome comparison between tetraploid wild wheat Zavitan and hexaploid bread wheat cv. Chinese Spring, aided by chromosome assemblies.

**Figure S4** Numbers of putative miRNA families identified in chromosome assemblies.

**Figure S5** Putative tRNA genes identified from repeat‐masked (top panel) and unmasked (bottom panel) chromosome assemblies.

**Figure S6.** A schematic representation of BTR alleles identified in the Zavitan genome and our chromosome assemblies.

**Figure S7.** Proposed model for autophagy induction in response to stress and developmental regulations.

**Table S1** *T. dicoccoides* contigs matching conserved grass genes and putatively wheat specific genes (excluding potential pseudogenes).

**Table S2** Summary of the single nucleotide polymorphisms and small InDels between *T. dicoccoides* survey sequences and pseudomolecules from cv. Zavitan.

**Table S3** Putative miRNAs encoded by *T. dicoccoides* chromosomes.

**Table S4** lncRNAs commonly mapping to chromosome assemblies, in addition to Zavitan and *T. aestivum* genomes. Potential lncRNA‐miRNA interactions are also included.

**Table S5** Overview of annotated ATG proteins from tetraploid and hexaploid wheat.

**Table S6** Simple Sequence Repeats (SSRs) and TE junctions found in chromosome assemblies that contain coding regions for selected agronomically important traits.

**Data S1** Selected agronomically important genes covered by chromosome assemblies.

**Data S2** Chromosome sorting, sequencing and assembly.

Supplementary File

## References

[pbi12940-bib-0001] Akpinar, B.A. and Budak, H. (2016) Dissecting miRNAs in wheat D genome progenitor, Aegilops tauschii. Front. Plant Sci. 7, 1–17. 27200073 10.3389/fpls.2016.00606 PMC4855405

[pbi12940-bib-0002] Akpinar, B.A. , Lucas, S.J. , Vr, J. , Dole, J. and Budak, H. (2014) Sequencing chromosome 5D of Aegilops tauschii and comparison with its allopolyploid descendant bread wheat (Triticum aestivum). Plant Biotechnol. J. 13, 740–752. 25516153 10.1111/pbi.12302

[pbi12940-bib-0003] Akpinar, B.A. , Kantar, M. and Budak, H. (2015a) Root precursors of microRNAs in wild emmer and modern wheats show major differences in response to drought stress. Funct. Integr. Genomics, 15, 587–598. 26174050 10.1007/s10142-015-0453-0

[pbi12940-bib-0004] Akpinar, B.A. , Yuce, M. , Lucas, S. , Vrána, J. , Burešová, V. , Doležel, J. and Budak, H. (2015b) Molecular organization and comparative analysis of chromosome 5B of the wild wheat ancestor Triticum dicoccoides. Sci. Rep. 5, 10763. 26084265 10.1038/srep10763 PMC4471722

[pbi12940-bib-0005] Akpinar, B.A. , Lucas, S. and Budak, H. (2016) A large‐scale chromosome‐specific SNP discovery guideline. Funct. Integr. Genomics, 17, 97–105. 27900504 10.1007/s10142-016-0536-6

[pbi12940-bib-0006] Alptekin, B. , Akpinar, B.A. and Budak, H. (2017) A comprehensive prescription for plant miRNA identification. Front. Plant Sci. 7, 2058. 28174574 10.3389/fpls.2016.02058 PMC5258749

[pbi12940-bib-0007] Avni, R. , Nave, M. , Barad, O. , Baruch, K. , Twardziok, S.O. , Gundlach, H. , Hale, I. *et al*. (2017) Wild emmer genome architecture and diversity elucidate wheat evolution and domestication. Science, 357, 93–97. 28684525 10.1126/science.aan0032

[pbi12940-bib-0008] Berkman, P.J. , Skarshewski, A. , Lorenc, M.T. , Lai, K. , Duran, C. , Ling, E.Y. , Stiller, J. *et al*. (2011) Sequencing and assembly of low copy and genic regions of isolated Triticum aestivum chromosome arm 7DS. Plant Biotechnol. J. 9, 768–775. 21356002 10.1111/j.1467-7652.2010.00587.x

[pbi12940-bib-0009] Berkman, P.J. , Lai, K. , Lorenc, M.T. and Edwards, D. (2012a) Next‐generation sequencing applications for wheat crop improvement. Am. J. Bot. 99, 365–371. 22268223 10.3732/ajb.1100309

[pbi12940-bib-0010] Berkman, P.J. , Skarshewski, A. , Manoli, S. , Lorenc, M.T. , Stiller, J. , Smits, L. , Lai, K. *et al*. (2012b) Sequencing wheat chromosome arm 7BS delimits the 7BS/4AL translocation and reveals homoeologous gene conservation. Theor. Appl. Genet. 124, 423–432. 22001910 10.1007/s00122-011-1717-2

[pbi12940-bib-0011] Budak, H. , Akpinar, B.A. , Unver, T. and Turktas, M. (2013a) Proteome changes in wild and modern wheat leaves upon drought stress by two‐dimensional electrophoresis and nanoLC‐ESI‐MS/MS. Plant Mol. Biol. 83, 89–103. 23443681 10.1007/s11103-013-0024-5

[pbi12940-bib-0012] Budak, H. , Kantar, M. and Kurtoglu, K.Y. (2013b) Drought tolerance in modern and wild wheat. Scientific World J. 2013, 548246. 10.1155/2013/548246 PMC3671283 23766697

[pbi12940-bib-0013] Cagirici, H.B. , Alptekin, B. and Budak, H. (2017) RNA sequencing and co‐expressed long non‐coding RNA in modern and wild wheats. Sci. Rep. 7, 10670. 28878329 10.1038/s41598-017-11170-8 PMC5587677

[pbi12940-bib-0014] Camacho, C. , Coulouris, G. , Avagyan, V. , Ma, N. , Papadopoulos, J. , Bealer, K. and Madden, T.L. (2009) BLAST+: architecture and applications. BMC Bioinform. 10, 421. 10.1186/1471-2105-10-421 PMC2803857 20003500

[pbi12940-bib-0015] Chikhi, R. and Medvedev, P. (2014) Informed and automated k‐mer size selection for genome assembly. Bioinformatics, 30, 31–37. 23732276 10.1093/bioinformatics/btt310

[pbi12940-bib-0016] Choulet, F. , Wicker, T. , Rustenholz, C. , Paux, E. , Salse, J. , Leroy, P. , Schlub, S. *et al*. (2010) Megabase level sequencing reveals contrasted organization and evolution patterns of the wheat gene and transposable element spaces. Plant Cell, 22, 1686–1701. 20581307 10.1105/tpc.110.074187 PMC2910976

[pbi12940-bib-0017] Choulet, F. , Alberti, A. , Theil, S. , Glover, N. , Barbe, V. , Daron, J. , Pingault, L. *et al*. (2014) Structural and functional partitioning of bread wheat chromosome 3B. Science, 345, 1249721. 25035497 10.1126/science.1249721

[pbi12940-bib-0018] Conesa, A. and Götz, S. (2008) Blast2GO: A comprehensive suite for functional analysis in plant genomics. Int. J. Plant Genomics, 2008, 619832. 18483572 10.1155/2008/619832 PMC2375974

[pbi12940-bib-0019] Distelfeld, A. (2016) Assembly and Validation of the Wild Emmer Wheat Genome. In Plant and Animal Genome XXIV Conference. Plant and Animal Genome. Available at: https://pag.confex.com/pag/xxiv/webprogram/Paper18477.html [Accessed May 11, 2016].

[pbi12940-bib-0020] Distelfeld, A. , Tranquilli, G. , Li, C. , Yan, L. and Dubcovsky, J. (2009) Genetic and molecular characterization of the VRN2 loci in tetraploid wheat. Plant Physiol. 149, 245–257. 19005084 10.1104/pp.108.129353 PMC2613703

[pbi12940-bib-0021] Ergen, N.Z. and Budak, H. (2009) Sequencing over 13 000 expressed sequence tags from six subtractive cDNA libraries of wild and modern wheats following slow drought stress. Plant, Cell Environ. 32, 220–236. 19054353 10.1111/j.1365-3040.2008.01915.x

[pbi12940-bib-0022] Ergen, N.Z. , Thimmapuram, J. , Bohnert, H.J. and Budak, H. (2009) Transcriptome pathways unique to dehydration tolerant relatives of modern wheat. Funct. Integr. Genomics, 9, 377–396. 19330365 10.1007/s10142-009-0123-1

[pbi12940-bib-0023] Feuillet, C. , Leach, J.E. , Rogers, J. , Schnable, P.S. and Eversole, K. (2011) Crop genome sequencing: lessons and rationales. Trends Plant Sci. 16, 77–88. 21081278 10.1016/j.tplants.2010.10.005

[pbi12940-bib-0024] Hao, M. , Luo, J. , Zhang, L. , Yuan, Z. , Zheng, Y. , Zhang, H. and Liu, D. (2013) In situ hybridization analysis indicates that 4AL‐5AL‐7BS translocation preceded subspecies differentiation of Triticum turgidum. Genome, 56, 303–305. 23789999 10.1139/gen-2013-0049

[pbi12940-bib-0025] Henry, R.J. , Rangan, P. and Furtado, A. (2016) Functional cereals for production in new and variable climates. Curr. Opin. Plant Biol. 30, 11–18. 26828379 10.1016/j.pbi.2015.12.008

[pbi12940-bib-0026] Hernandez, P. , Martis, M. , Dorado, G. , Pfeifer, M. , Gálvez, S. , Schaaf, S. , Jouve, N. *et al*. (2012) Next‐generation sequencing and syntenic integration of flow‐sorted arms of wheat chromosome 4A exposes the chromosome structure and gene content. Plant J. 69, 377–386. 21974774 10.1111/j.1365-313X.2011.04808.x

[pbi12940-bib-0027] Iizumi, T. , Yokozawa, M. , Sakurai, G. , Travasso, M.I. , Romanenkov, V. , Oettli, P. , Newby, T. *et al*. (2014) Historical changes in global yields: major cereal and legume crops from 1982 to 2006. Glob. Ecol. Biogeogr. 23, 346–357.

[pbi12940-bib-0028] Jia, J. , Zhao, S. , Kong, X. , Li, Y. , Zhao, G. , He, W. , Appels, R. *et al*. (2013) Aegilops tauschii draft genome sequence reveals a gene repertoire for wheat adaptation. Nature, 496, 91–95. 23535592 10.1038/nature12028

[pbi12940-bib-0029] Kantar, M. , Akpınar, B.A. , Valárik, M. , Lucas, S.J. , Doležel, J. , Hernández, P. and Budak, H. (2012) Subgenomic analysis of microRNAs in polyploid wheat. Funct. Integr. Genomics, 12, 465–479. 22592659 10.1007/s10142-012-0285-0

[pbi12940-bib-0030] Klionsky, D. , Agholme, L. , Agnello, M. , Agostinis, P. , Aguirre‐Ghiso, J.A. , Ahn, H.J. , Ait‐Mohamed, O. *et al*. (2016) Guidelines for the use and interpretation of assays for monitoring autophagy. Autophagy, 8, 445–544. 10.4161/auto.19496 PMC3404883 22966490

[pbi12940-bib-0031] Krzywinski, M. , Schein, J. , Birol, I. , Connors, J. , Gascoyne, R. , Horsman, D. , Jones, S.J. *et al*. (2009) Circos: an information aesthetic for comparative genomics. Genome Res. 19, 1639–1645. 19541911 10.1101/gr.092759.109 PMC2752132

[pbi12940-bib-0032] Kurtoglu, K.Y. , Kantar, M. , Lucas, S.J. and Budak, H. (2013) Unique and conserved microRNAs in wheat chromosome 5D revealed by next‐generation sequencing. PLoS ONE, 8, e69801. 23936103 10.1371/journal.pone.0069801 PMC3720673

[pbi12940-bib-0033] Kurtoglu, K.Y. , Kantar, M. and Budak, H. (2014) New wheat microRNA using whole‐genome sequence. Funct. Integr. Genomics, 14, 363–379. 24395439 10.1007/s10142-013-0357-9

[pbi12940-bib-0034] Kuzuoglu‐Ozturk, D. , Cebeci Yalcinkaya, O. , Akpinar, B.A. , Mitou, G. , Korkmaz, G. , Gozuacik, D. and Budak, H. (2012) Autophagy‐related gene, TdAtg8, in wild emmer wheat plays a role in drought and osmotic stress response. Planta, 236, 1081–1092. 22569921 10.1007/s00425-012-1657-3

[pbi12940-bib-0035] Li, L. , Stoeckert, C.J. and Roos, D.S. (2003) OrthoMCL: identification of ortholog groups for eukaryotic genomes. Genome Res. 13, 2178–2189. 12952885 10.1101/gr.1224503 PMC403725

[pbi12940-bib-0036] Li, F. , Pignatta, D. , Bendix, C. , Brunkard, J.O. , Cohn, M.M. , Tung, J. and Sun, H. (2011a) MicroRNA regulation of plant innate immune receptors. Proc. Natl. Acad. Sci. USA, 109, 1790–1795. 10.1073/pnas.1118282109 PMC3277104 22307647

[pbi12940-bib-0037] Li, Y. , Li, C. , Xia, J. and Jin, Y. (2011b) Domestication of transposable elements into MicroRNA genes in plants. PLoS ONE, 6, e19212. 21559273 10.1371/journal.pone.0019212 PMC3086885

[pbi12940-bib-0038] Ling, H.‐Q. , Zhao, S. , Liu, D. , Wang, J. , Sun, H. , Zhang, C. , Fan, H. *et al*. (2013) Draft genome of the wheat A‐genome progenitor Triticum urartu. Nature, 496, 87–90. 23535596 10.1038/nature11997

[pbi12940-bib-0039] Liu, H. , Searle, I.R. , Watson‐Haigh, N.S. , Baumann, U. , Mather, D.E. , Able, A.J. *et al*. (2015) Genome‐wide identification of MicroRNAs in leaves and the developing head of four durum genotypes during water deficit stress T. Unver, ed. PLoS ONE, 10, e0142799. 26562166 10.1371/journal.pone.0142799 PMC4643036

[pbi12940-bib-0040] Lowe, T.M. and Eddy, S.R. (1997) tRNAscan‐SE: A program for improved detection of transfer RNA genes in genomic sequence. Nucleic Acids Res. 25, 0955–0964. 10.1093/nar/25.5.955 PMC146525 9023104

[pbi12940-bib-0041] Lucas, S.J. and Budak, H. (2012) Sorting the wheat from the chaff: identifying miRNAs in genomic survey sequences of Triticum aestivum chromosome 1AL. A. A. Aboobaker, ed. PLoS ONE, 7, e40859. 22815845 10.1371/journal.pone.0040859 PMC3398953

[pbi12940-bib-0042] Lucas, S.J. , Akpinar, B.A. , Šimková, H. , Kubaláková, M. , Doležel, J. and Budak, H. (2014) Next‐generation sequencing of flow‐sorted wheat chromosome 5D reveals lineage‐specific translocations and widespread gene duplications. BMC Genom. 15, 1–18. 10.1186/1471-2164-15-1080 PMC4298962 25487001

[pbi12940-bib-0043] Luo, M. , Gu, Y.Q. , You, F.M. , Deal, K.R. , Ma, Y. , Hu, Y. *et al*. (2013) A 4‐gigabase physical map unlocks the structure and evolution of the complex genome of Aegilops tauschii, the wheat D‐genome progenitor. Proc. Natl. Acad. Sci. USA, 110, 7940–7945. 23610408 10.1073/pnas.1219082110 PMC3651469

[pbi12940-bib-0044] Marcussen, T. , Sandve, S.R. , Heier, L. , Spannagl, M. , Pfeifer, M. , International Wheat Genome Sequencing Consortium , Jakobsen, K.S. *et al*. (2014) Ancient hybridizations among the ancestral genomes of bread wheat. Science, 345, 1250092. 25035499 10.1126/science.1250092

[pbi12940-bib-0045] Mayer, K.F. , Waugh, R. , Brown, J.W. , Schulman, A. , Langridge, P. , Platzer, M. , Fincher, G.B. *et al*. (2012) A physical, genetic and functional sequence assembly of the barley genome. Nature, 491, 711–716. 23075845 10.1038/nature11543

[pbi12940-bib-0046] Mayer, K.F. , Rogers, J. , Doležel, J. , Pozniak, C. , Eversole, K. , Feuillet, C. , Gill, B. *et al*. (2014) A chromosome‐based draft sequence of the hexaploid bread wheat (Triticum aestivum) genome. Science, 345, 1251788. 25035500 10.1126/science.1251788

[pbi12940-bib-0047] Nagy, P. , Hegedus, K. , Pircs, K. , Varga, Á. and Juhász, G. (2014) Different effects of Atg2 and Atg18 mutations on Atg8a and Atg9 trafficking during starvation in Drosophila. FEBS Lett. 588, 408–413. 24374083 10.1016/j.febslet.2013.12.012 PMC3928829

[pbi12940-bib-0048] Nevo, E. and Chen, G. (2010) Drought and salt tolerances in wild relatives for wheat and barley improvement. Plant, Cell Environ. 33, 670–685. 20040064 10.1111/j.1365-3040.2009.02107.x

[pbi12940-bib-0049] Nussbaumer, T. , Martis, M.M. , Roessner, S.K. , Pfeifer, M. , Bader, K.C. , Sharma, S. , Gundlach, H. *et al*. (2013) MIPS PlantsDB: a database framework for comparative plant genome research. Nucleic Acids Res. 41, D1144–D1151. 23203886 10.1093/nar/gks1153 PMC3531202

[pbi12940-bib-0050] Paterson, A.H. , Bowers, J.E. , Bruggmann, R. , Dubchak, I. , Grimwood, J. , Gundlach, H. , Haberer, G. *et al*. (2009) The Sorghum bicolor genome and the diversification of grasses. Nature, 457, 551–556. 19189423 10.1038/nature07723

[pbi12940-bib-0051] Paux, E. , Faure, S. , Choulet, F. , Roger, D. , Gauthier, V. , Martinant, J.P. , Sourdille, P. *et al*. (2010) Insertion site‐based polymorphism markers open new perspectives for genome saturation and marker‐assisted selection in wheat. Plant Biotechnol. J. 8, 196–210. 20078842 10.1111/j.1467-7652.2009.00477.x

[pbi12940-bib-0052] Pei, D. , Zhang, W. , Sun, H. , Wei, X. , Yue, J. and Wang, H. (2014) Identification of autophagy‐related genes ATG4 and ATG8 from wheat (Triticum aestivum L.) and profiling of their expression patterns responding to biotic and abiotic stresses. Plant Cell Rep. 33, 1697–1710. 24996626 10.1007/s00299-014-1648-x

[pbi12940-bib-0053] Pourkheirandish, M. , Hensel, G. , Kilian, B. , Senthil, N. , Chen, G. , Sameri, M. , Azhaguvel, P. *et al*. (2015) Evolution of the grain dispersal system in barley. Cell, 162, 527–539. 26232223 10.1016/j.cell.2015.07.002

[pbi12940-bib-0054] Sahoo, R.K. , Gill, S.S. and Tuteja, N. (2012) Pea DNA helicase 45 promotes salinity stress tolerance in IR64 rice with improved yield. Plant Signal. Behav. 7, 1042–1046. 22827940 10.4161/psb.20915 PMC3474676

[pbi12940-bib-0055] Sela, H. , Spiridon, L.N. , Petrescu, A.J. , Akerman, M. , Mandel‐Gutfreund, Y. , Nevo, E. , Loutre, C. *et al*. (2012) Ancient diversity of splicing motifs and protein surfaces in the wild emmer wheat (Triticum dicoccoides) LR10 coiled coil (CC) and leucine‐rich repeat (LRR) domains. Mol. Plant Pathol. 13, 276–287. 21952112 10.1111/j.1364-3703.2011.00744.x PMC6638671

[pbi12940-bib-0056] Simpson, J.T. , Wong, K. , Jackman, S.D. , Schein, J.E. , Jones, S.J.M. and Birol, I. (2009) ABySS: a parallel assembler for short read sequence data. Genome Res. 19, 1117–1123. 19251739 10.1101/gr.089532.108 PMC2694472

[pbi12940-bib-0057] Sun, L. , Luo, H. , Bu, D. , Zhao, G. , Yu, K. , Zhang, C. , Liu, Y. *et al*. (2013) Utilizing sequence intrinsic composition to classify protein‐coding and long non‐coding transcripts. Nucleic Acids Res. 41, e166. 23892401 10.1093/nar/gkt646 PMC3783192

[pbi12940-bib-0058] Tanaka, T. , Antonio, B.A. , Kikuchi, S. , Matsumoto, T. , Nagamura, Y. , Numa, H. , Sakai, H. *et al*. (2008) The rice annotation project database (RAP‐DB): 2008 update. Nucleic Acids Res. 36, D1028–D1033. 18089549 10.1093/nar/gkm978 PMC2238920

[pbi12940-bib-0059] Tanaka, T.S. , Kobayashi, F.U. , Joshi, G.I.R.I.P.R. , Onuki, R. , Sakai, H. , Kanamori, H. , Wu, J. *et al*. (2013) Next‐generation survey sequencing and the molecular organization of wheat chromosome 6B. DNA Res., 21, 103–114. 24086083 10.1093/dnares/dst041 PMC3989483

[pbi12940-bib-0060] The International Brachypodium Initiative (2010) Genome sequencing and analysis of the model grass Brachypodium distachyon. Nature, 463, 763–768. 20148030 10.1038/nature08747

[pbi12940-bib-0061] Tuteja, N. (2010) A Method to Confer Salinity Stress Tolerance to Plants by Helicase Overexpression. In Helicases. pp. 377–387. Available at: http://link.springer.com/10.1007/978-1-60327-355-8. 10.1007/978-1-60327-355-8_26 20225163

[pbi12940-bib-0062] Vatansever, R. , Filiz, E. and Eroglu, S. (2017) Genome‐wide exploration of metal tolerance protein (MTP) genes in common wheat (Triticum aestivum): insights into metal homeostasis and biofortification. Biometals, 30, 217–235. 28150142 10.1007/s10534-017-9997-x

[pbi12940-bib-0063] Venora, G. , Blangiforti, S. , Castiglione, M.R. , Pignone, D. , Losavio, F. and Cremonini, R. (2002) Chromatin organisation and computer aided karyotyping of Triticum durum Desf. cv. Timilia. Caryologia, 55, 91–98.

[pbi12940-bib-0064] Vitulo, N. , Albiero, A. , Forcato, C. , Campagna, D. , Dal Pero, F. , Bagnaresi, P. , Colaiacovo, M. *et al*. (2011) First survey of the wheat chromosome 5A composition through a next generation sequencing approach. E. Newbigin, ed. PLoS ONE, 6, e26421. 22028874 10.1371/journal.pone.0026421 PMC3196578

[pbi12940-bib-0065] Wu, T.D. and Watanabe, C.K. (2005) GMAP: a genomic mapping and alignment program for mRNA and EST sequences. Bioinformatics, 21, 1859–1875. 15728110 10.1093/bioinformatics/bti310

[pbi12940-bib-0066] Xie, W. and Nevo, E. (2008) Wild emmer: genetic resources, gene mapping and potential for wheat improvement. Euphytica, 164, 603–614.

[pbi12940-bib-0067] Yan, L. (2004) The wheat VRN2 gene is a flowering repressor down‐regulated by vernalization. Science, 303, 1640–1644. 15016992 10.1126/science.1094305 PMC4737501

[pbi12940-bib-0068] Yue, J. , Sun, H. , Zhang, W. , Pei, D. , He, Y. and Wang, H. (2015) Wheat homologs of yeast ATG6 function in autophagy and are implicated in powdery mildew immunity. BMC Plant Biol. 15, 1–15. 25888209 10.1186/s12870-015-0472-y PMC4393579

[pbi12940-bib-0069] Zhai, J. , Jeong, D.‐H. , De Paoli, E. , Park, S. , Rosen, B.D. , Li, Y. , González, A.J. *et al*. (2011) MicroRNAs as master regulators of the plant NB‐LRR defense gene family via the production of phased, trans‐acting siRNAs. Genes Dev. 25, 2540–2553. 22156213 10.1101/gad.177527.111 PMC3243063

[pbi12940-bib-0070] Zhu, Q.H. , Fan, L. , Liu, Y. , Xu, H. , Llewellyn, D. and Wilson, I. (2013) miR482 regulation of NBS‐LRR defense genes during fungal pathogen infection in cotton. PLoS ONE, 8, e84390. 24391949 10.1371/journal.pone.0084390 PMC3877274

[pbi12940-bib-0071] Zimin, A.V. , Puiu, D. , Hall, R. , Kingan, S. , Clavijo, B.J. and Salzberg, S.L. (2017) The first near‐complete assembly of the hexaploid bread wheat genome, Triticum aestivum. Gigascience, 6, 1–7. 10.1093/gigascience/gix097 PMC5691383 29069494

