# Supplementary figures and images for: Chromosome‐based survey sequencing reveals the genome organization of wild wheat progenitor Triticum dicoccoides
Source: Plant Biotechnol J. 2018 Jun 13;16(12):2077–87. doi: 10.1111/pbi.12940 (PMC6230948; doi:10.1111/pbi.12940)

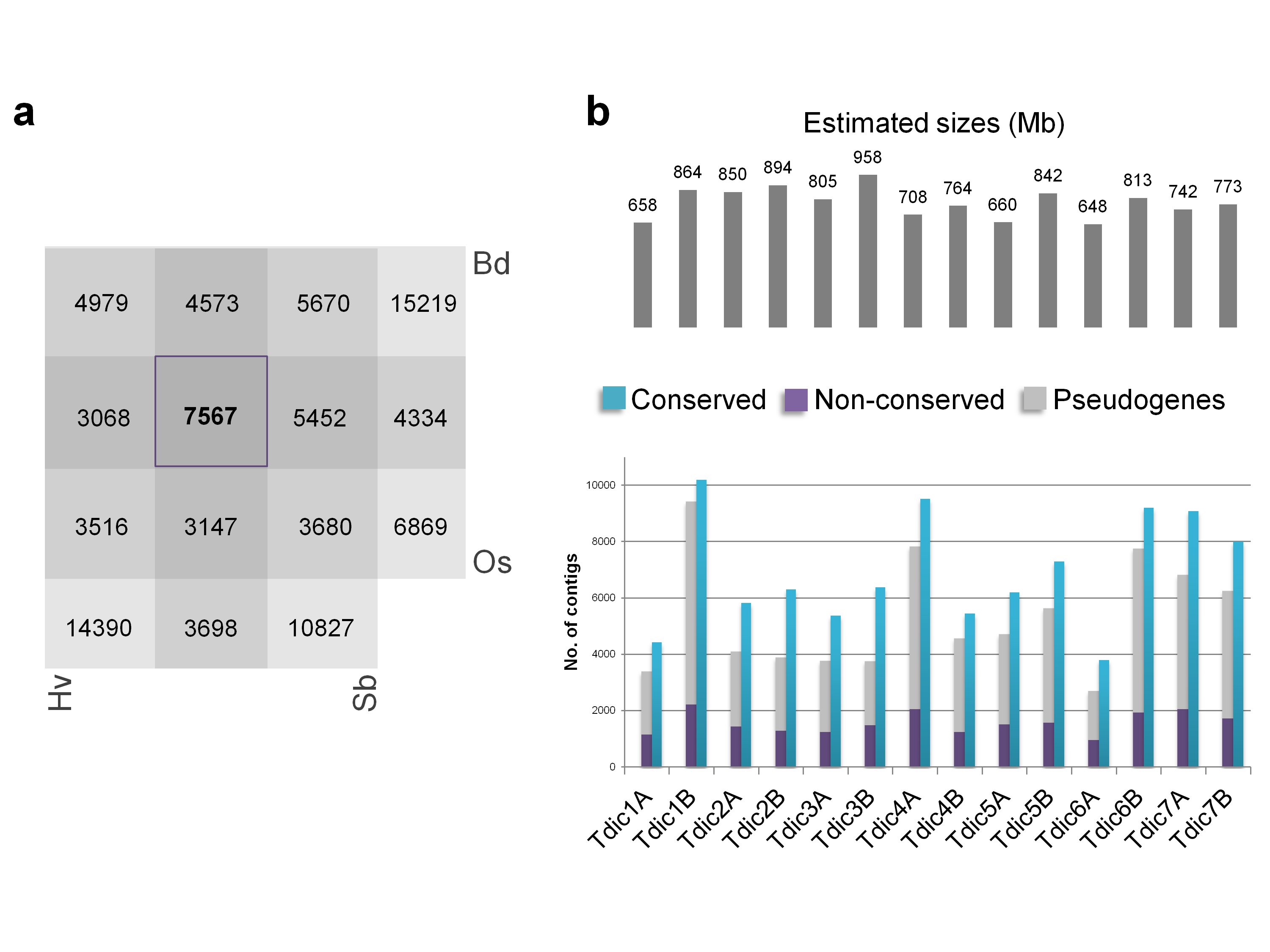

Supplement: Supplementary file 1 — Figure S1 Sequences from chromosome assemblies associated with conserved and non‐conserved genes. [file PBI-16-2077-s015.tif]

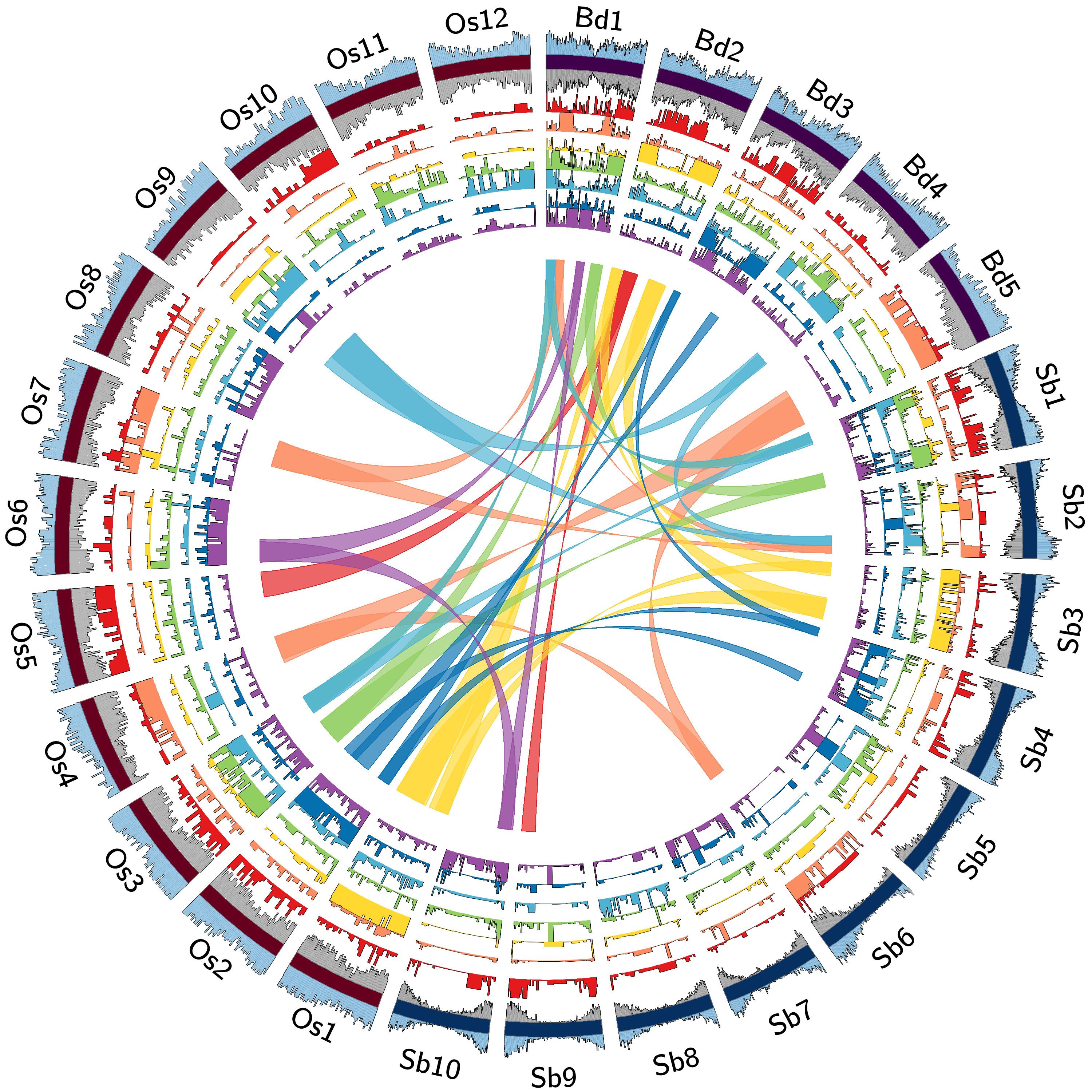

Supplement: Supplementary file 2 — Figure S2 Syntenic relationships between T. dicoccoides and related grasses Brachypodium (Bd), rice (Os) and sorghum (Sb). [file PBI-16-2077-s016.png]

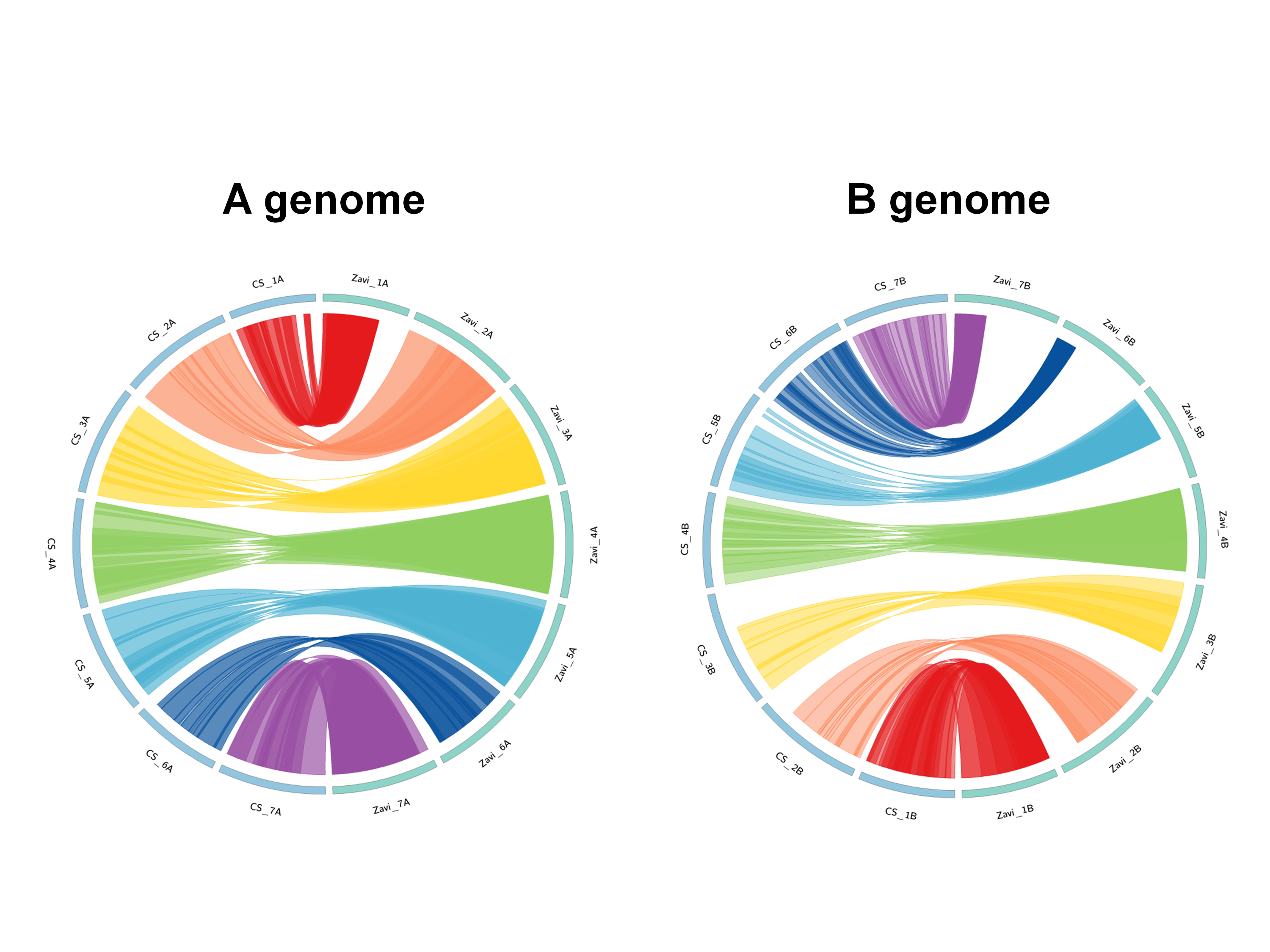

Supplement: Supplementary file 3 — Figure S3 Overview of the structural genome comparison between tetraploid wild wheat Zavitan and hexaploid bread wheat cv. Chinese Spring, aided by chromosome assemblies. [file PBI-16-2077-s014.tif]

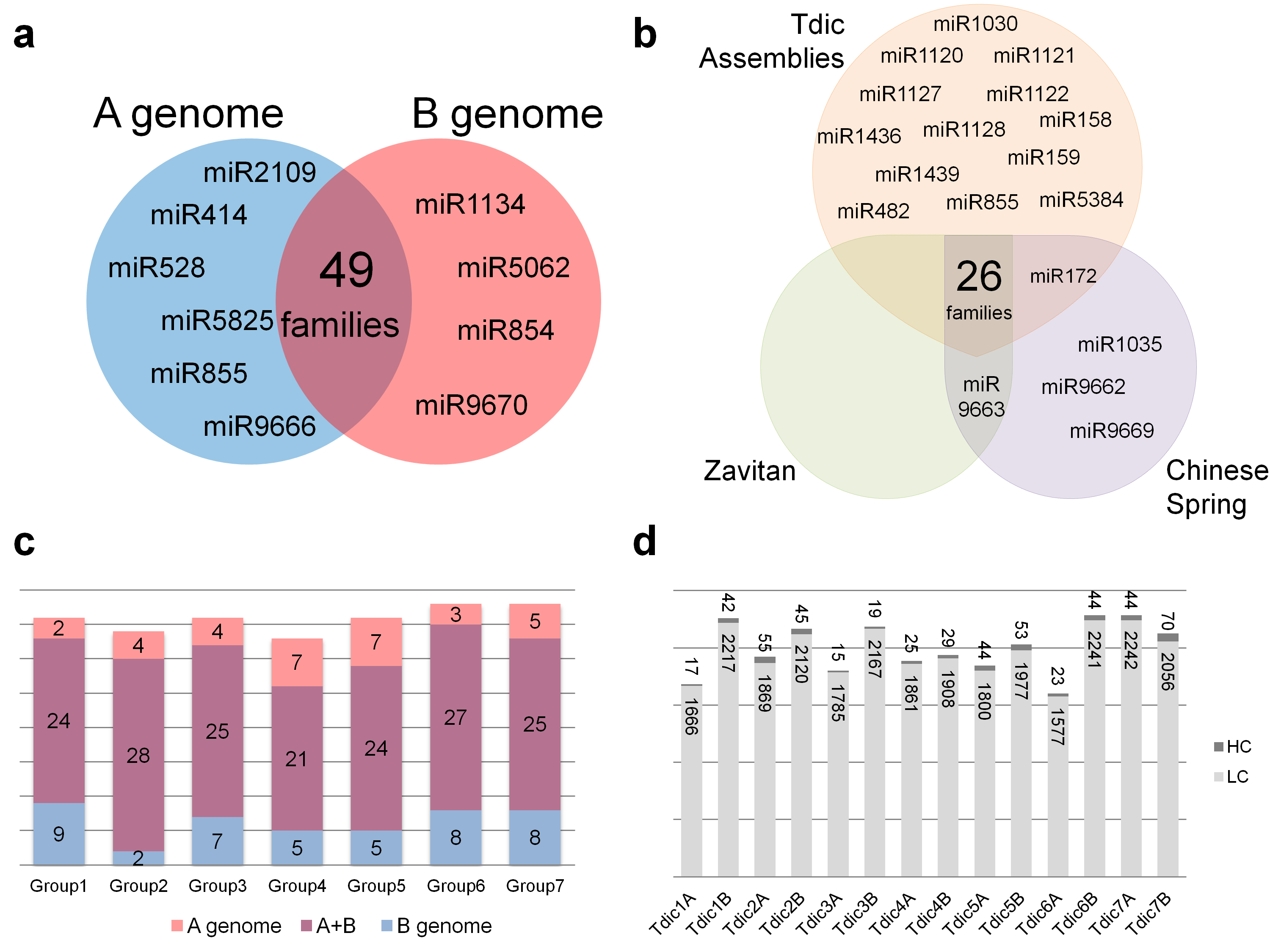

Supplement: Supplementary file 4 — Figure S4 Numbers of putative miRNA families identified in chromosome assemblies. [file PBI-16-2077-s013.tif]

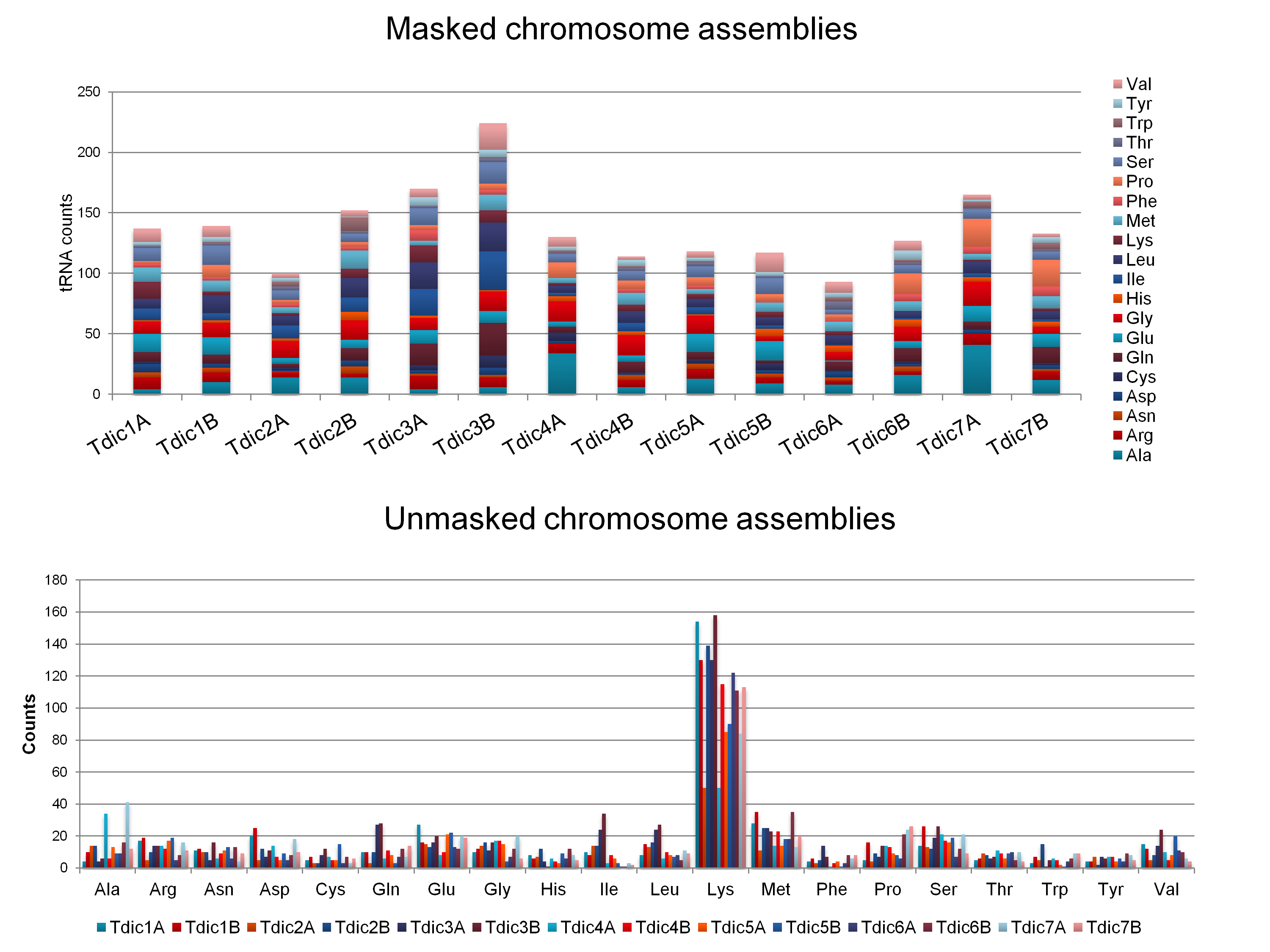

Supplement: Supplementary file 5 — Figure S5 Putative tRNA genes identified from repeat‐masked (top panel) and unmasked (bottom panel) chromosome assemblies. [file PBI-16-2077-s012.tif]
