# Supplementary material for: Chromosome‐based survey sequencing reveals the genome organization of wild wheat progenitor Triticum dicoccoides
Source: Plant Biotechnol J. 2018 Jun 13;16(12):2077–87. doi: 10.1111/pbi.12940 (PMC6230948; doi:10.1111/pbi.12940)

Supplementary Figure S6. A schematic representation of BTR alleles identified in the Zavitan genome and our chromosome assemblies.

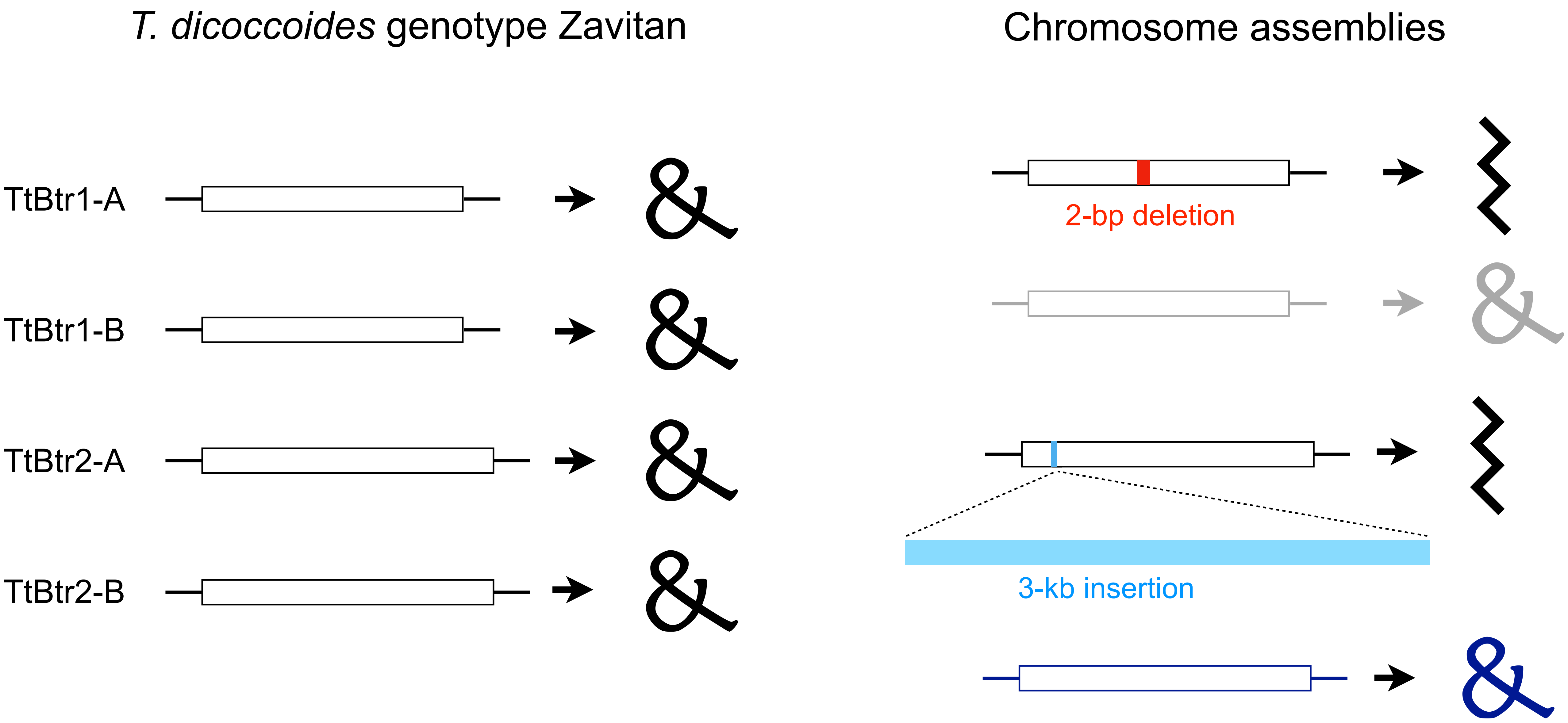

Supplement: Supplementary file 6 — Figure S6. A schematic representation of BTR alleles identified in the Zavitan genome and our chromosome assemblies. [file PBI-16-2077-s001.pdf]
