# Supplementary material for: Chromosome‐based survey sequencing reveals the genome organization of wild wheat progenitor Triticum dicoccoides
Source: Plant Biotechnol J. 2018 Jun 13;16(12):2077–87. doi: 10.1111/pbi.12940 (PMC6230948; doi:10.1111/pbi.12940)

Supplementary Figure S7. Proposed model for autophagy induction in response to stress and developmental regulations. Hormone and stress responsive transcription factors binds to promotor region of ATG genes and induce the autophagy based on gene expression and ATG protein translation. Autophagy generate rapid energy production based on the fast turnover of old proteins and organelles, thus help cell to maintain stability under stress conditions. It also promotes cell differentiation and help plant development and reproduction.

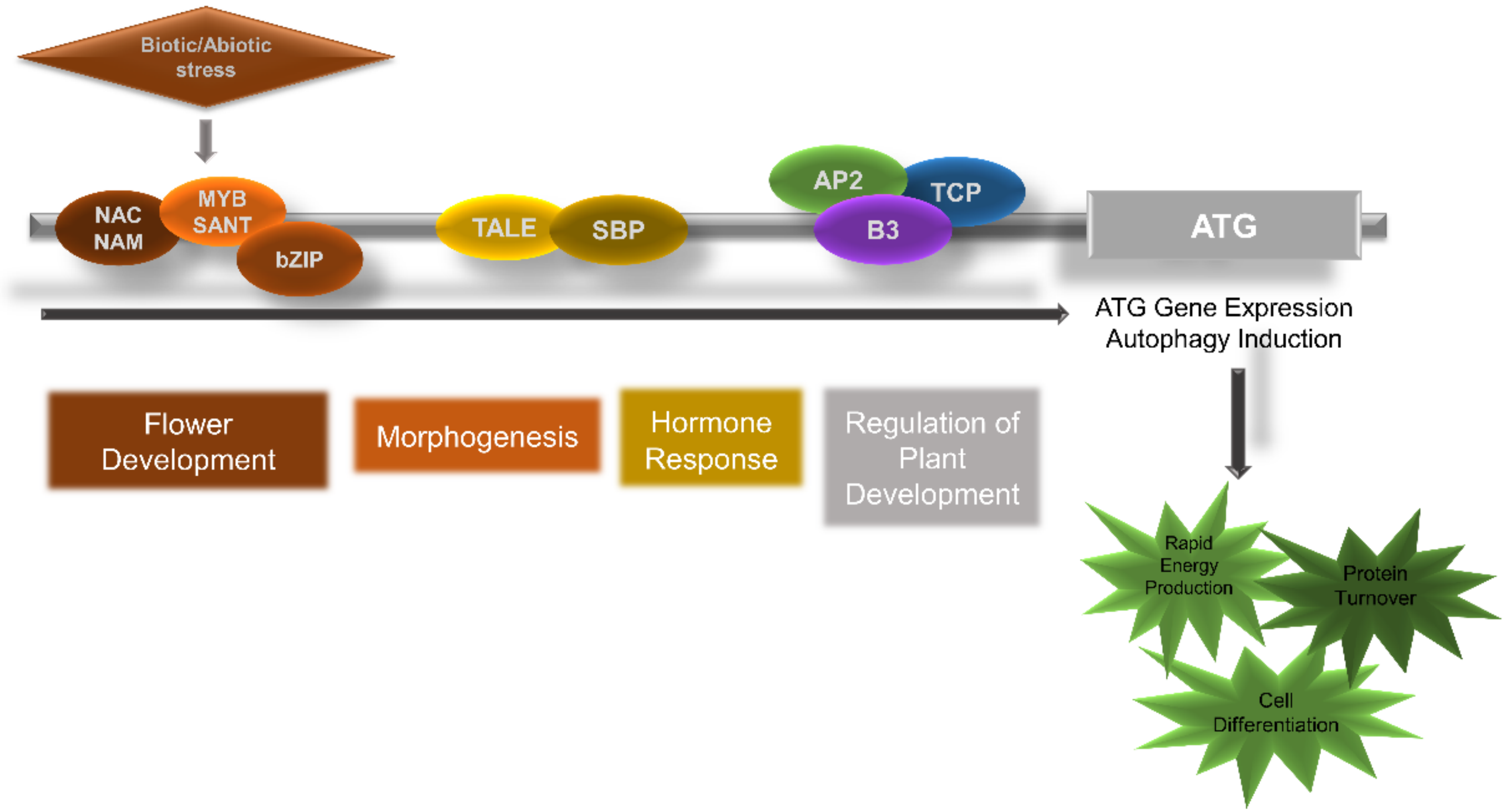

Supplement: Supplementary file 7 — Figure S7. Proposed model for autophagy induction in response to stress and developmental regulations. [file PBI-16-2077-s002.pdf]
