# Supplementary material for: Chromosome‐based survey sequencing reveals the genome organization of wild wheat progenitor Triticum dicoccoides
Source: Plant Biotechnol J. 2018 Jun 13;16(12):2077–87. doi: 10.1111/pbi.12940 (PMC6230948; doi:10.1111/pbi.12940)
Supplement: Supplementary file 9 — Table S2 Summary of the single nucleotide polymorphisms and small InDels between T. dicoccoides survey sequences and pseudomolecules from cv. Zavitan. [file PBI-16-2077-s004.docx]

**Supplementary Table S2. Summary of the single nucleotide polymorphisms and small InDels between *T. dicoccoides* survey sequences and pseudomolecules from cv. Zavitan.**

| Characteristic | *T. dicoccoides* survey sequences |
| --- | --- |
| Mean depth^1^ | 47.8× |
| Total no. of SNPs^2^ | 42,747,789 |
| Total no. of InDels^2^ | 2,256,544 |
| Mean SNPs/kb^2^ | 4.18 |
| Ti/Tv^2^ |  |
| Tdic1A | 2.59 |
| Tdic1B | 2.27 |
| Tdic2A | 2.52 |
| Tdic2B | 2.36 |
| Tdic3A | 2.54 |
| Tdic3B | 2.38 |
| Tdic4A | 2.30 |
| Tdic4B | 2.30 |
| Tdic5A | 2.55 |
| Tdic5B | 2.38 |
| Tdic6A | 2.53 |
| Tdic6B | 2.20 |
| Tdic7A | 2.37 |
| Tdic7B | 2.27 |

^1^Mean depth was calculated based on the raw sequence read data given in Table 1.

^2^Calculated via comparison to pseudomolecule sequences from cv. Zavitan.

Ti: transitions; Tv: transversions.
