# Supplementary material for: Chromosome‐based survey sequencing reveals the genome organization of wild wheat progenitor Triticum dicoccoides
Source: Plant Biotechnol J. 2018 Jun 13;16(12):2077–87. doi: 10.1111/pbi.12940 (PMC6230948; doi:10.1111/pbi.12940)
Supplement: Supplementary file 12 — Table S5 Overview of annotated ATG proteins from tetraploid and hexaploid wheat. [file PBI-16-2077-s007.docx]

**Supplementary Table S5. Overview of annotated ATG proteins from tetraploid and hexaploid wheat.**

|  | Located Chromosome |  | Orthologs of wheat ATGs proteins |
| --- | --- | --- | --- |

| **Protein Name** | **Hexaploid Wheat** | **Tetraploid Wheat** | **Associated Domain** | **Arabidopsis** | **Rice** | **Brachypodium** |
| --- | --- | --- | --- | --- | --- | --- |
| ATG2 | 7A, 7B, 7D | 7A, 7B | ATG_C domain | 36% | 71% | 82% |
| ATG3 | 1A, 1B, 1D | 1A, 1B | Autophagy_N, Autophagy_act_C, Autophagy_C | 72% | 89% | 90% |
| ATG4a/b | 2A, 2D, Un | 2A, 2B | Peptidase_C54 | 54% | 83% | 83% |
| ATG5 | 6B, Un | 6A, 6B | APG5 superfamily domain | 52% | 77% | 78% |
| ATG6 | 3B, 3D | 3A, 3B | APG6 superfamily domain | 59% | 87% | 96% |
| ATG7 | 3A, 3B, 3D | 3A, 3B | ATG7_N superfamily domain, Ubiquitin-activating enzyme (E1 enzyme) domain | 47% | 74% | 82% |
| ATG8a-f | 2A, 2B, 2D | 2A, 2B | ATG8 super family domain | 88% | 96% | 97% |
| ATG8g | 2A, 2B, 2D | 2A, 2B | ATG8 super family domain | 71% | 94% | 97% |
| ATG8h | 5A, 5D | 5A, 5B | ATG8 super family domain | 61% | 89% | 92% |
| ATG9 | 4A, 4B, 4D | 4A, 4B | Apg9 superfamily domain | 53% | 80% | 87% |
| ATG10 | 2A, 2B, 2D | 2A, 2B | Autophagy_act_C domain | 46% | 70% | 75% |
| ATG12 | 6A, 6D | 6A, 6B | APG12 superfamily domain | 75% | 76% | 86% |
