# Supplementary material for: Chromosome‐based survey sequencing reveals the genome organization of wild wheat progenitor Triticum dicoccoides
Source: Plant Biotechnol J. 2018 Jun 13;16(12):2077–87. doi: 10.1111/pbi.12940 (PMC6230948; doi:10.1111/pbi.12940)
Supplement: Supplementary file 14 — Data S1 Selected agronomically important genes covered by chromosome assemblies. [file PBI-16-2077-s009.docx]

**Supplementary Data S1.** Selected agronomically important genes covered by chromosome assemblies.

*T. dicoccoides* chromosome assemblies cover several agronomically important traits related to domestication, nutrient quality and stress responses, demonstrating the utility of these assemblies. For domestication-related traits, besides the *Btr* alleles, *T. dicoccoides* chromosome assemblies covered homeologous coding regions for the Q-locus (spelt factor), which controls domestication-related traits, free threshing, rachis fragility and spike shape and has a pleiotropic effect on other domestication-related traits. The recessive allele (q), with non-free-threshing, ‘spelt’ spikes and fragile rachises, differs from the dominant allele (Q) by a single amino acid substitution at the 329^th^ position of the translated product and by the absence of a putative miRNA172 binding site at exon 10 (Sormacheva *et al.*, 2015). Tdic5A-contig17630980 included 81% of the 1344-nucleotide coding sequence (9 of the 10 exons). Both the genomic and coding sequences shared exceptionally high sequence identity (over 99%), and the translated product (identical to the *T. dicoccoides* Q-protein, AFV46177.1) contained the characteristic 329V residue for spelt spikes. Consistent with previous observations, a gene homeologous at both the nucleotide and amino acid levels to the Q-gene and protein, but with several substitutions, was found on chromosome 5B Cluster_542517; this likely represents a pseudogene that can still affect the spelt phenotype (Sormacheva *et al.*, 2015).

Glutenins and gliadins are major storage proteins in wheat seeds. The High Molecular Weight Glutenin subunits (HMW-GSs), which affect dough strength and processing quality through determining gluten elasticity, exhibit high allelic diversity, particularly in tetraploid species(Jiang *et al.*, 2012). Contig6436197 and contig6381928 from Tdic1A harbored coding regions for x-type and y-type HMW-GSs, respectively, likely representing the *Glu-A1* locus, including its upstream and downstream regions. The 27-kb contig6436197 contained a single exon between positions 14,570 and 17,065 that was translated into an 830-residue protein product with the characteristic N and C termini and the repetitive central domain. This protein was highly similar to x-type subunits identified previously (Jiang *et al.*, 2012) and had seven substitutions compared to the closest known homolog (AHZ62762.1). The 596 amino acid-protein encoded by the 12-kb contig6381928, from positions 3819 to 5612, had y-type homologs and a deletion spanning six amino acids, in addition to five substitutions, compared to the closest y-type subunit identified thus far (Jiang *et al.*, 2012). Inactivation of *1Ax* and/or *1Ay* HMW-GSs, primarily due to the presence of premature stop codons, results in the expression of one or a few HMW-GSs (Jiang *et al.*, 2012). No premature stop codons were detected for the protein products from contig6314147 and contig6381928, suggesting that both alleles are functional and expressed. Importantly, both contigs spanned the entire coding region, including the upstream promoter and downstream sequences. Five more contigs from Tdic1A had potential coding regions for gliadins. Although three of these contigs contained only partial sequences, internal stop codons and/or extensive rearrangements, suggesting they corresponded to pseudogenes, open reading frames (ORFs) on contig6181486 (positions 2335–3243) and contig6442657 (positions 4087–4977) could be translated into 285 and 296 amino acid peptides, respectively, with significant similarities to known gliadins. γ-gliadins can contain several epitopes that trigger autoimmune responses in people with celiac disease and are thus the most toxic components of wheat gluten. Therefore, alleles for shorter γ-gliadins, particularly in the repetitive domain, might represent a safer alternative for wheat improvement (Qi *et al.*, 2009).

Micronutrients are vitally important for both plant development and grain quality. Analysis of the chromosome assemblies of *T. dicoccoides* hinted at the presence of a few transporters important for metal homeostasis. Since the key players of metal ion uptake and transport in wheat are generally unknown or poorly characterized at the genome level, we used the sequences of well-characterized homologous proteins from rice to identify highly similar coding regions, particularly on syntenic wheat chromosomes. Three contigs, from Tdic1A and Tdic1B, namely Tdic1A-contig6381675, Tdic1B-contig28073667 and Tdic1B-contig28051069, contained coding regions for potential homologs of OsMTP1 (Metal Tolerance Protein, UniProt Q688R1). OsMTP1 is a Zn transporter encoded by a gene located on syntenic rice chromosome 5. Their syntenic relationships, the presence of intact cation efflux family domains and the high sequence similarity (>98%) to *Ae. tauschii* MTP1 protein and barley Zn transporters suggested these regions might encode *bona fide* Zn transporters. Although additional regions on syntenic Tdic4A, Tdic4B and Tdic6B highly similar to *OsMTP2* and *OsMTP3* were identified, a closer look revealed that they only partially covered the respective proteins, suggest that these copies correspond to pseudogenes.

Another protein related to Zn metabolism, ZRT/IRT-like protein 4 (BAH93579.1) encoded by one gene on rice chromosome 6, matched contig20520127 from the syntenic Tdic7A chromosome, ; this gene was translatinged into a short peptide (as is the rice protein) with a well-defined Zip domain. A second contig, contig20761513 from Tdic7A, shared relatively low similarity with rice ZRT/IRT-like protein 4, but it still contained a Zip domain. Contig4767398 and contig17228122 from Tdic6A and Tdic6B, respectively, had coding regions highly similar to *OsYSL15*, which plays important roles in iron uptake and transport; this gene is located on syntenic rice chromosome 2(Inoue *et al.*, 2009). Two more contigs on Tdic6A and Tdic6B also had potential coding regions with relatively low sequence similarity to *OsYSL15* (BAG88718.1). Whether these sequences represent new players in the Graminaceae-specific iron uptake route (Masuda *et al.*, 2017) awaits further analysis.

Two contigs from the Tdic2A assembly contained sequences that were translated into full-length peptides highly similar to sulfate/molybdate transporters of the sulfate uptake pathway, which is at least partially overlapping with the selenium uptake pathway(Shinmachi *et al.*, 2010). Finally, Tdic7B-contig12593266 contained coding regions for a potential boron transporter. These findings demonstrate that the *T. dicoccoides* chromosome assemblies provide a rich resource for exploring and exploiting the allelic diversity of key transporter proteins for micronutrient uptake and distribution.

Another major gene affecting flowering in wheat, *Ppd1*, was potentially encoded by an ORF located within contig17452792 from Tdic2A. The protein product translated by this 4.7 kb-long contig was identical to the pseudo-response regulator protein expressed from the *Ppd-A1* locus (BAL63553.1). These observations indicate that our chromosome assemblies cover most of the genetic switches that regulate flowering in wheat.

Leaf rust caused by *Puccinia triticina* is an important disease that affects wheat production worldwide (Sela *et al.*, 2012; Xie and Nevo, 2008). A leaf rust resistance gene, *Lr10*, was located inside the 13-kb contig6213438 from Tdic1A. The *Lr10* coding sequence within this contig was composed of two exons and one intron, encoding a 929 amino-acid protein product. Pfam and sequence similarity searches revealed three functional domains spanning amino acids 11–133 (CC-like), 180–465 (NB-ARC) and 586–697 (LRR), which is typical of *Lr* genes. The coding sequence for *Lr10* was located between nucleotides 4817 and 8775 on the 13 kb-long contig6213438, allowing us to identify the closely linked *RGA2* gene (GenBank: EU675963.1). Comparisons with *Lr10* sequences from 58 *T. dicoccoides* accessions (Sela *et al.*, 2011, 2012) indicated that they share 94–98% sequence identity, with the highest identity corresponding to the intron regions. The LR10 protein encoded by contig6213438 and the LR10 proteins expressed by the 58 *T. dicoccoides* accessions differed considerably, especially in the CC (coiled-coil) domain, which is consistent with previous observations, including InDels and amino acid substitutions(Sela *et al.*, 2011, 2012). Compared with the closest protein product from the 58 accessions, the LR10 protein from contig6213438 had a two-amino acid insertion at the 279^th^ position, raising the possibility of a new allele for the *Lr10* locus.

In a recent study, a major QTL, *QHf.osu-1A* for Hessian fly resistance, was found to account for 70% of the phenotypic variability in hexaploid wheat (Tan *et al.*, 2013). BAC sequencing to further dissect this QTL to identify candidate resistance genes revealed a cluster composed of four OPR genes encoding proteins in the jasmonic acid biosynthesis pathway, a stress-induced receptor-like kinase (Srk), catalytic domain of protein kinases (Pkc) and a calcineurin-like phosphoesterase-like protein (Clp). Three contigs in our 1A chromosome assembly, contig6444640, contig6367519 and contig6370545, covered the *Srk*, *Pkc* and *Clp* genes, respectively, whereas Tdic1A-contig6361585 and Tdic1A_Cluster_3673 contained coding regions for the OPRs.

In addition to these closely spaced biotic stress-related genes on chromosome 1AS, the *T. dicoccoides* chromosome assemblies covered other biotic stress-associated loci, such as *Tsn1* for tan spot disease, in addition to several abiotic stress-related loci, including general stress-responsive TF genes such as MYB/MYC and WRKY family genes and genes encoding general stress-responsive proteins such as heat shock proteins, late embryogenesis abundant proteins, several drought-responsive dehydrins and homeologous copies of the cold-responsive TF WCOR15. Additionally, the coding regions for high-affinity K^+^ transporters (HKTs), which might play a role in salt transport, were also identified in our chromosome assemblies. Taken together, these results indicate that the *T. dicoccoides* chromosome assemblies cover the gene-rich regions of the genome quite well.

**References**

Inoue, H., Kobayashi, T., Nozoye, T., Takahashi, M., Kakei, Y., Suzuki, K., et al. (2009) *Rice OsYSL15 is an iron-regulated iron(III)-deoxymugineic acid transporter expressed in the roots and is essential for iron uptake in early growth of the seedlings.* *J. Biol. Chem.*, **284**, 3470–9.

Jiang, Q.T., Ma, J., Zhao, S., Zhao, Q.Z., Lan, X.J., Dai, S.F., et al. (2012) *Characterization of HMW-GSs and their gene inaction in tetraploid wheat*. *Genetica*, **140**, 325–335.

Masuda, H., Shimochi, E., Hamada, T., Senoura, T., Kobayashi, T., Aung, M.S., et al. (2017) *A new transgenic rice line exhibiting enhanced ferric iron reduction and phytosiderophore production confers tolerance to low iron availability in calcareous soil*. *PLoS One*, **12**, e0173441.

Qi, P.-F., Wei, Y.-M., Ouellet, T., Chen, Q., Tan, X., and Zheng, Y.-L. (2009) *The gamma-gliadin multigene family in common wheat (Triticum aestivum) and its closely related species.* *BMC Genomics*, **10**, 168.

Sela, H., Loutre, C., Keller, B., Schulman, A., Nevo, E., Korol, A., and Fahima, T. (2011) *Rapid linkage disequilibrium decay in the Lr10 gene in wild emmer wheat (Triticum dicoccoides) populations*. *Theor. Appl. Genet.*, **122**, 175–187.

Sela, H., Spiridon, L.N., Petrescu, A.J., Akerman, M., Mandel-Gutfreund, Y., Nevo, E., et al. (2012) *Ancient diversity of splicing motifs and protein surfaces in the wild emmer wheat (Triticum dicoccoides) LR10 coiled coil (CC) and leucine-rich repeat (LRR) domains*. *Mol. Plant Pathol.*, **13**, 276–287.

Shinmachi, F., Buchner, P., Stroud, J.L., Parmar, S., Zhao, F.-J., McGrath, S.P., and Hawkesford, M.J. (2010) *Influence of sulfur deficiency on the expression of specific sulfate transporters and the distribution of sulfur, selenium, and molybdenum in wheat.* *Plant Physiol.*, **153**, 327–336.

Sormacheva, I., Golovnina, K., Vavilova, V., Kosuge, K., Watanabe, N., Blinov, A., and Goncharov, N.P. (2015) *Q gene variability in wheat species with different spike morphology*. *Genet. Resour. Crop Evol.*, **62**, 837–852.

Tan, C.T., Carver, B.F., Chen, M.-S., Gu, Y.-Q., and Yan, L. (2013) *Genetic association of OPR genes with resistance to Hessian fly in hexaploid wheat.* *BMC Genomics*, **14**, 369.

Xie, W. and Nevo, E. (2008) *Wild emmer: genetic resources, gene mapping and potential for wheat improvement*. *Euphytica*, **164**, 603–614.
