# Supplementary material for: Chromosome‐based survey sequencing reveals the genome organization of wild wheat progenitor Triticum dicoccoides
Source: Plant Biotechnol J. 2018 Jun 13;16(12):2077–87. doi: 10.1111/pbi.12940 (PMC6230948; doi:10.1111/pbi.12940)
Supplement: Supplementary file 15 — Data S2 Chromosome sorting, sequencing and assembly. [file PBI-16-2077-s010.docx]

**Chromosome-based survey sequencing reveals the genome organization of wild wheat progenitor *Triticum dicoccoides***

Bala Ani Akpinar^1^, Sezgi Biyiklioglu^1^, Burcu Alptekin^1^, Miroslava Havránková^2^, Jan Vrána^2^, Jaroslav Doležel^2^, Assaf Distelfeld^3^, Pilar Hernandez^4^, The IWGSC^5^, Hikmet Budak^1*^

**Supplementary Data S2.**

**Chromosome sorting, sequencing and assembly**

Bivariate flow karyotyping and chromosome sorting were carried out as previously described (Akpinar *et al.*, 2015). Aqueous suspensions of intact mitotic metaphase chromosomes were prepared from primary roots of germinating seeds. Prior to cytometric analysis, fluorescence *in situ* hybridization in suspension (FISHIS) was performed on a suspension of isolated chromosomes using oligonucleotide 5ʹ-FITC-GAA7-FITC-3ʹ to improve chromosome resolution. After FISHIS, the chromosomes were stained with DAPI (2 μg/ml) and the samples were analyzed using FACSAria SORP (BD Biosciences, San Jose, USA) at rate of 1,500 chromosomes/sec. Blue laser (488 nm, 100 mW) was used to excite FITC fluorescence of the GAA microsatellites, and UV laser (355 nm, 100 mW) was used for DAPI excitation. Biparametric flow karyotypes of FITC fluorescence (logarithmic scale) and DAPI fluorescence (linear scale) were obtained after analyzing 20,000 chromosomes and used to identify populations of individual chromosomes. To assess the purity of the sorted fractions, three batches of 2,000 chromosomes were sorted into a drop of P5 buffer on a microscopic slide and air-dried. The sorted chromosomes were identified by microscopic observation after fluorescence *in situ* hybridization (FISH) using probes for GAA microsatellites and Afa repeats, which resulted in chromosome-specific labeling patterns.

For each chromosome, three batches of ~30,000 chromosomes, equivalent to 50 ng of chromosomal DNA, were sorted into a PCR tube with 40 μl deionized water, and the chromosomal DNA was amplified by isothermal multiple displacement amplification (MDA), yielding 7.78–12.56 µg of DNA for each chromosome for direct sequencing.

**Clustering of grass proteins into orthologous groups**

In total, 135,705 grass proteins from (1) *Brachypodium distachyon* (v1.2, <http://mips.helmholtz-muenchen.de/plant/brachypodium>) (The International Brachypodium Initiative, 2010), (2) *Oryza sativa* (IRGSP-1.0, <http://rapdb.dna.affrc.go.jp/download/irgsp1.html>) (Tanaka *et al.*, 2008), (3) *Sorghum bicolor* (v1.4, <http://mips.helmholtz-muenchen.de/plant/sorghum/>) (Paterson *et al.*, 2009) (1E-6, -length 30, -ppos 75) and (4) *Hordeum vulgare* proteins (high confidence proteins, <http://mips.helmholtz-muenchen.de/plant/barley/>) (Mayer *et al.*, 2012) (1E-6, -length 30, -ppos 90) were clustered into 21,385 orthologous groups containing 110,114 proteins. The longest sequences were selected as representatives of these orthologous groups. The 25,591 remaining proteins that could not be allocated to orthologous groups and thus were left as singletons were added to the list of orthologous group representatives, generating a final list of 46,976 ‘non-redundant’ orthologous grass proteins. This list was used to estimate the number of conserved genes encoded by *T. dicoccoides* chromosomes by performing BLAST searches for sequence similarity. Significant hits with *T. dicoccoides* contigs were identified with the parameters and cutoffs given above. Additionally, all full-length coding sequences for *T. dicoccoides* and *T. turgidum* were retrieved from NCBI (<http://www.ncbi.nlm.nih.gov/>) to search for genes entirely covered by *T. dicoccoides* contigs.

**Homology-based *in silico* miRNA identification**

A homology-based miRNA prediction approach was utilized to annotate miRNA-encoding sequences as described previously, with slight modifications (Alptekin *et al.*, 2017; Kurtoglu *et al.*, 2014, 2013; Lucas and Budak, 2012). Briefly, two in-house Perl scripts, SUmirFind and SUmirFold, were used to identify contigs with high sequence similarity to 1,404 previously known mature miRNA sequences (high confidence and experimental) and to evaluate the secondary structure characteristics of the predicted precursor miRNA (pre-miRNA) sequences based on previously established criteria, respectively (Kurtoglu *et al.*, 2013; Lucas and Budak, 2012). Previously identified mature miRNA sequences for *Viridiplantae* species were obtained from miRBase Release 21 (<http://www.mirbase.org/>). Only miRNAs with direct experimental evidence or miRNAs deemed to be ‘high-confidence’ in the miRBase database were included in the miRNA prediction pipeline. Expression analysis of the predicted miRNAs was performed at both the pre-miRNA and mature miRNA level. At the pre-miRNA level, all potential miRNA precursor sequences were compared against a) transcriptome assemblies also used in target identification, b) EST sequences deposited in NCBI dbEST (<http://www.ncbi.nlm.nih.gov/dbEST/>, 9343 entries as of 22.08.16) and c) wheat EST assemblies deposited in PlantGDB EST (<http://www.plantgdb.org/>, 8,513 entries) via BLAST searches. Precursor sequences matching known EST or transcript sequences at 95% sequence identity over at least 95% of their length were considered to be ‘expressed’. At the mature miRNA level, all predicted mature miRNA sequences were compared against durum wheat small RNA sequences generated in a recent study (Liu *et al.*, 2015). The quality of the raw sequence reads was checked using FASTQC, and adapters were removed using Cutadapt software (<https://github.com/marcelm/cutadapt>, -e 0 -O 10 -m 30 --max-n 0.1) (Martin, 2011). Sequence comparisons were carried out using the blastn tool of the BLAST+ package (-task blastn -short -ungapped -dust 'no' -evalue 1000 -strand 'plus' -perc_identity 100 -word_size 7 -qcov_hsp_perc 100). Repetitive sequences within putative pre-miRNA sequences were identified using RepeatMasker software with an updated TE database (Daron *et al.*, 2014), and pre-miRNA sequences covered by repetitive elements more than 50% of their lengths were retrieved and analyzed using custom scripts.

**Identification of autophagy-related genes**

To generate the construct for ATG proteins, the reviewed ATG protein sequences from SwissProt database (<https://web.expasy.org/docs/swiss-prot_guideline.html>) were searched and, Arabidopsis and Rice ATG proteins were listed. To identify the ATG homologs from wheat, listed protein sequences were searched against EST sequences using Wheat EST assemblies and several wheat RNA-Seq assemblies listed above were used through BLAST. For homology search, reciprocal best hit approach was utilized with conduction of both blastx (evalue: 1e – 6) and tblastn (evalue: 1e – 5) analysis. Blast results were filtered based on ppos:50 and alignment length:30 parameters and the sequences which provide these parameters in both blastx and tblastn analysis were extracted and assembled into contigs via VectorNTI.11.5. Assembled contigs were translated into proteins via Expasy translator (<http://web.expasy.org/translate/>) all 6 frames. The produced protein sequences were blasted to whole NR protein db at NCBI website and their homology to known ATG proteins from Brachypodium, Rice and Arabidopsis was detected. mRNA constructs were mapped to wheat genome version 1 assembly to identify exon-intron boundaries with gmap program (-f gff_gene, --exon_genomic, -Q). The gff results were used to generate gene models via GSDS server (<http://gsds.cbi.pku.edu.cn/>). The exon sequences were manually merged to detect the mRNA sequences from genomic region. The mRNA sequences were manually controlled to see if they provide the correct protein sequences with a proper start and stop codon. The cis element and promotor region analysis of ATG genes was conducted with PlantPan 2.0 (<http://plantpan2.itps.ncku.edu.tw/>) by taking 1 kb upstream of defined ATG genes. The expression analysis of ATG genes were conducted using WheatExp (<https://wheat.pw.usda.gov/WheatExp/>) after homology search of ATG genes against deposited RNA-Seq data (blast evalue: 1e-10).

**References**

Akpinar, B.A., Yuce, M., Lucas, S., Vrána, J., Burešová, V., Doležel, J., and Budak, H. (2015) *Molecular organization and comparative analysis of chromosome 5B of the wild wheat ancestor Triticum dicoccoides.* *Sci. Rep.*, **5**, 10763.

Alptekin, B., Akpinar, B.A., and Budak, H. (2017) *A comprehensive prescription for plant miRNA identification*. *Front. Plant Sci.*, **7**.

Daron, J., Glover, N., Pingault, L., Theil, S., Jamilloux, V., Paux, E., et al. (2014) *Organization and evolution of transposable elements along the bread wheat chromosome 3B*. *Genome Biol.*, **15**, 546.

Kurtoglu, K.Y., Kantar, M., and Budak, H. (2014) *New wheat microRNA using whole-genome sequence.* *Funct. Integr. Genomics*.

Kurtoglu, K.Y., Kantar, M., Lucas, S.J., and Budak, H. (2013) *Unique and conserved microRNAs in wheat chromosome 5D revealed by next-generation sequencing.* *PLoS One*, **8**, e69801.

Liu, H., Searle, I.R., Watson-Haigh, N.S., Baumann, U., Mather, D.E., Able, A.J., et al. (2015) *Genome-Wide Identification of MicroRNAs in Leaves and the Developing Head of Four Durum Genotypes during Water Deficit Stress*. *PLoS One*, **10**, e0142799.

Lucas, S.J. and Budak, H. (2012) *Sorting the wheat from the chaff: identifying miRNAs in genomic survey sequences of Triticum aestivum chromosome 1AL.* *PLoS One*, **7**, e40859.

Martin, M. (2011) *Cutadapt removes adapter sequences from high-throughput sequencing reads*. *EMBnet.journal*, **17**, 10–12.

Mayer, K.F.X., Waugh, R., Langridge, P., Close, T.J., Wise, R.P., Graner, A., et al. (2012) *A physical, genetic and functional sequence assembly of the barley genome*. *Nature*, **491**, 711–716.

Paterson, A.H., Bowers, J.E., Bruggmann, R., Dubchak, I., Grimwood, J., Gundlach, H., et al. (2009) *The Sorghum bicolor genome and the diversification of grasses.* *Nature*, **457**, 551–6.

Tanaka, T., Antonio, B.A., Kikuchi, S., Matsumoto, T., Nagamura, Y., Numa, H., et al. (2008) *The Rice Annotation Project Database (RAP-DB): 2008 update.* *Nucleic Acids Res.*, **36**, D1028-33.

The International Brachypodium Initiative (2010) *Genome sequencing and analysis of the model grass Brachypodium distachyon.* *Nature*, **463**, 763–768.
